# Decoding the Postulated Entourage Effect of Medicinal *Cannabis*: What It Is and What It Isn’t

**DOI:** 10.3390/biomedicines11082323

**Published:** 2023-08-21

**Authors:** Catalina Christensen, Martin Rose, Claus Cornett, Morten Allesø

**Affiliations:** 1 Tetra Pharm Technologies ApS, Rugmarken 10, DK-3650 Ølstykke, Denmark; martin@tetrapharm.eu (M.R.); morten@tetrapharm.eu (M.A.); 2 Department of Pharmacy, University of Copenhagen, Universitetsparken 2, DK-2100 Copenhagen, Denmark; claus.cornett@sund.ku.dk

**Keywords:** entourage effect, synergy, bioenhancer, antagonism, drug–drug interaction, polypharmacology, polypharmacy, medicinal cannabis, cannabinoids, active pharmaceutical ingredient

## Abstract

The ‘entourage effect’ term was originally coined in a pre-clinical study observing endogenous bio-inactive metabolites potentiating the activity of a bioactive endocannabinoid. As a hypothetical afterthought, this was proposed to hold general relevance to the usage of products based on *Cannabis sativa* L. The term was later juxtaposed to polypharmacy pertaining to full-spectrum medicinal *Cannabis* products exerting an overall higher effect than the single compounds. Since the emergence of the term, a discussion of its pharmacological foundation and relevance has been ongoing. Advocates suggest that the ‘entourage effect’ is the reason many patients experience an overall better effect from full-spectrum products. Critics state that the term is unfounded and used primarily for marketing purposes in the *Cannabis* industry. This scoping review aims to segregate the primary research claiming as well as disputing the existence of the ‘entourage effect’ from a pharmacological perspective. The literature on this topic is in its infancy. Existing pre-clinical and clinical studies are in general based on simplistic methodologies and show contradictory findings, with the clinical data mostly relying on anecdotal and real-world evidence. We propose that the ‘entourage effect’ is explained by traditional pharmacological terms pertaining to other plant-based medicinal products and polypharmacy in general (e.g., synergistic interactions and bioenhancement).

## 1. Introduction—The Emergence of the ‘Entourage Effect’ Term 

Ever since the discovery of the endocannabinoid system (ECS) in 1988, the scientific community has had a strong interest in exploring the therapeutic potential of *Cannabis sativa* L. (hereafter referred to as “*Cannabis*”). The ECS is almost ubiquitously distributed throughout the body and as such is implicated in maintaining homeostasis across multiple physiological functions. It is often observed as being either up- or downregulated in different disease states. Targeting this system with ECS modulatory compounds, e.g., exogenous *Cannabis*-derived cannabinoids (e.g., Δ^9^-tetrahydrocannabinol (THC) and cannabidiol (CBD)) can aid in rebalancing the system to homeostasis, resulting in therapeutic effects. It is generally recognized that the system is composed of cannabinoid receptors (e.g., cannabinoid receptor 1 (CB1) and cannabinoid receptor 2 (CB2)); endocannabinoid signaling molecules (e.g., anandamide (AEA) and 2-arachidonoylglycerol (2-AG)); and enzymes responsible for the metabolism and availability of endocannabinoids. These ECS components are regulated in response to disturbances in the homeostasis of various body systems at any given time [1]. 

To the best of our knowledge, the ‘entourage effect’ term was used for the first time in a pre-clinical study performed by Ben-Shabat et al. in 1998 [2]. They found that endogenous metabolites (i.e., fatty acid glycerol esters), which are otherwise individually pharmacologically inactive, potentiated the activity of the endocannabinoid 2-AG when tested collectively in different in vitro and in vivo studies. The potentiated effect was only observed at specific metabolite concentration ranges. This was as a hypothetical afterthought described as the ‘entourage effect’ and proposed to potentially be of broad relevance to the medicinal use of *Cannabis*-based products. The authors referred to bioactive compounds derived from plants as being accompanied by chemically related compounds (i.e., ‘entourage compounds’), the latter being bio-inactive when administered individually. The observations by Ben-Shabat et al. could therefore potentially lead to findings suggesting that plant products exert effects that resemble those found in nature more than isolated single compounds from the plant [2,3]. 

The application of the ‘entourage effect’ term in the literature was later on juxtaposed with polypharmacy more broadly, pertaining to full-spectrum medicinal *Cannabis* products, exerting an overall higher effect compared to single compounds (e.g., THC and CBD) isolated from the plant or their synthetic analogues. Advocates of the term suggest the ‘entourage effect’ mechanism to be the underlying reason that many patients claim to experience an overall better effect from full-spectrum *Cannabis* products [4,5]. This, however, relies mostly on anecdotal and real-world evidence from observational studies. Critics, on the contrary, state that the ‘entourage effect’ is unsupported by sound evidence and that the term primarily is used for marketing purposes to promote sales in the currently blooming medicinal *Cannabis* industry [6,7]. 

The aim of this scoping review is to discuss the main literature claiming the existence of the ‘entourage effect’ and to decode the referenced evidence from a pharmacological angle. Research disputing the existence of the ‘entourage effect’ is also included to provide an objective point of view on the matter. The overall aim is to clarify if evidence-based rationales exist that justify the usage of the ‘entourage effect’ term when referring to a superior therapeutic effect of full-spectrum medicinal *Cannabis* products compared to single compound analogues. The review is based on literature searches performed on PubMed until April 2023, with the following combination of search terms: “entourage effect”, “synergy”, “full-spectrum”, and “medicinal cannabis”; or “medical cannabis”, “cannabinoid”, and “terpene”; or “terpenoid”, “herbal bioenhancer”, “polypharmacy”, and “polypharmacology”. Reference lists of key reviews and articles were assessed for additional articles of importance to the topic. Reviews, perspectives, and original research of both pre-clinical and clinical origin have been included.

## 2. Possible Pharmacological Mechanisms Involved in the ‘Entourage Effect’

To progress *Cannabis*-based products from their current status as medicinal *Cannabis* under national control to a conventional drug supported by clinical evidence and with centralized approval by, e.g., the EMA and FDA, it is necessary to adhere to scientific pharmacological terminology. A misleading tendency—describing the ‘entourage effect’ as synergistic effects arising between the *Cannabis*-derived cannabinoids and terpenes resulting in a collective potentiated beneficial therapeutic effect—is evident. This implies that the compounds act at the same receptor targets while avoiding the fact that antagonistic interactions might also arise. Decoding the proclaimed entourage effect with a pharmacological lens therefore seems warranted. 

### 2.1. Pharmacokinetic and Pharmacodynamic Interactions 

In recent decades, research has focused highly on discovering and determining the pharmacological effects involved in the ‘entourage effect’ [8]. The exact mechanisms of actions are still unknown; however, in general, they are believed to involve pharmacokinetic and pharmacodynamic interactions between the compounds of the *winterized* extract, that is, with plant waxes removed but cannabinoids and auxiliary compounds like terpenes and flavonoids preserved. Interactions occur when one compound’s activity is affected by other compounds either negatively or positively. Drug interactions can be of a pharmacokinetic and pharmacodynamic nature, which collectively can exert both beneficial and adverse clinical outcomes. Pharmacokinetic interactions affect the involved compounds’ absorption, distribution, metabolism, and excretion (ADME), ultimately impacting their bioavailability. Pharmacodynamic interactions between compounds of the full-spectrum extract affect the efficacy of the dosed medicine. These interactions happen due to differences in receptor/enzyme targets and/or binding affinities that either enhance or suppress the bioactivity of other involved compounds [9,10,11]. 

### 2.2. Additive, Synergistic, and Antagonistic Effects 

Multiple types of combinatory effects are possible between the *Cannabis* compounds, including additive effects and synergistic as well as antagonistic interactions [9]. Additive effects between compounds are a pure summation of the individual compounds’ effects. Antagonistic interactions result in a combinatory effect lower than the sum of individual effects. This is often considered negative in the context of therapeutic effect but can be beneficial if the dampened antagonized effect reduces unwanted adverse effects. Synergistic interactions are the result of two or more compounds working in concert to cause a potentiated effect greater than the sum of their individual effects [12]. The ‘entourage effect’ is often described as being caused by beneficial synergistic effects, while antagonistic effects or additive adverse effects often are avoided in the discussion [7]. Underlying mechanisms of actions resulting in such synergistic effects, in addition to the elimination of adverse effects, can, as mentioned above, be of pharmacokinetic and pharmacodynamic origin [12,13,14]. 

### 2.3. Bioenhancers

Bioavailability refers to the fraction of a drug that reaches the systemic circulation and ultimately its therapeutic target(s). Bioenhancers increase bioavailability, thus enabling the drug to reach its therapeutic response at a lower dose, carrying the additional benefit of reducing the likelihood of adverse effects [15]. For a bioactive compound to exert its full therapeutic potential, its absorption and resultant bioavailability is paramount. Depending on the route of administration, the oral route being the most restrictive, limitations in permeability, water solubility, and first-pass metabolism in the liver can decrease the compound’s bioavailability. Cannabinoids are intrinsically prone to poor bioavailability as a result of their lipophilic chemical nature and thus poor water solubility as well as the reported first-pass metabolism of both CBD and THC [16]. Bioenhancer mechanisms of actions can therefore involve, e.g., the enhancement of absorption, inhibition of drug efflux membrane transporters, and inhibition of cytochrome P-450 (CYP450) liver enzymes. Examples of bioenhancers of natural origin are, e.g., grapefruit juice, citric acid, aloe vera, flavonoid curcumin, menthol, and eicosapentaenoic fatty acid. These are, among many others, applied as excipients in different final drug products in traditional pharmacology [17,18]. 

These pharmacological mechanisms of action ultimately impact the compounds’ individual and collective effects and the resultant clinical outcome experienced by the patient. 

## 3. Proclaimed Entourage Compounds 

Following the original findings by Ben-Shabat et al., other studies have investigated different endocannabinoid-like compounds and referred to them as ‘entourage compounds’ when they were observed to potentiate the activity of the endocannabinoids 2-AG and AEA (e.g., [19,20,21,22]).

Two types of ‘entourage effects’ have been defined in relation to *Cannabis*-derived compounds: ‘intra-entourage’ and ‘inter-entourage’. The former refers to either cannabinoid-to-cannabinoid or terpene-to-terpene interactions, and the latter refers to cannabinoid-to-terpene interactions [23].

THC itself has been reported to be a partial agonist of both CB1 and CB2, in addition to possessing affinity and exerting effects at other targets, observed across numerous pre-clinical studies (Table A1). THC can exert mixed agonistic and antagonistic effects depending on different factors such as cell type, receptor expression state, and the presence of other ligands with affinity for the same targets as THC (e.g., endocannabinoids or other cannabinoids derived from the *Cannabis* plant). The concentration of THC in relation to other potentially co-administered compounds (i.e., ‘entourage compounds’) also impacts the pharmacological effect [24]. 

CBD binds to a multitude of targets (summarized in Table A1) and as such possesses a promiscuous and complex pharmacological profile [25]. The polypharmacology of CBD is under extensive investigation for a variety of postulated pharmacological effects across multiple pathologies (e.g., neurological, neuropsychiatric, and inflammatory disorders), explaining why more data are available on this compound [26,27]. Some of CBD’s mechanisms of action, such as binding to CB1 as an allosteric negative modulator [28], can impact the bioactivity of THC. This has led to the proposal that CBD is an ‘entourage compound’ [4,5,29]. CBD additionally can impact the pharmacokinetics of THC by, e.g., inhibiting some hepatic CYP enzymes. As such, the metabolism of THC into its more potent psychoactive metabolite (i.e., 11-OH-THC) will be delayed [30,31]. CBD furthermore can modulate endocannabinoid pharmacokinetics by inhibiting fatty acid amide hydrolase (FAAH), thus inhibiting the degradation of AEA [32]. 

Terpenes have also been referred to as ‘entourage compounds’ due to their ability to, e.g., increase blood–brain barrier permeability, thus increasing the pharmacokinetic properties of, e.g., THC [30,33]. A non-exhaustive list of pharmacological effects pertaining to terpenes is provided in Table A2. 

Flavonoids are another group of compounds present in *Cannabis*, and more than 20 different flavonoids have been identified [34]. The bioactivities and therapeutic potentials of these compounds have not yet been studied in depth. However, Cannaflavins A-C have been reported to possess anti-inflammatory, neuroprotective, anti-cancer, and anti-viral effects. These compounds are therefore likely to contribute to the collective therapeutic effect exerted by the administration of a *Cannabis*-derived extract and as such could be perceived as ‘entourage compounds’ too. 

The abovementioned observations, among others, have led to the proposal that THC can be perceived as a ‘silver bullet’, while the additional compounds derived from *Cannabis* can be perceived as a collective ‘synergistic shotgun’ [4,5,30]. 

## 4. Evidence Perceived as Supporting the ‘Entourage Effect’

In line with the original definition of the ‘entourage effect’, several endocannabinoid-like compounds have been investigated for their ‘entourage effect’ in relation to the endocannabinoids 2-AG and AEA. An ‘entourage effect’ of 2-AG and related endogenous compounds (2-linoleoylglycerol (2-LG) and 2-palmitoylglycerol (2-PG)) with an analgesic effect in cultured neurons was observed. A delay in transient receptor potential vanilloid 1 (TRPV1) signaling was seen when the compounds were administered in combination [19]. Another group of researchers [20] observed the same tendency, with AEA congeners (palmitoylethanolamide (PEA) and oleamide (OEA)) potentiating the vasorelaxant effect of AEA via TRPV1 targeting in rats. PEA and analogue compounds have been reported to act as ‘entourage compounds’ by preventing AEA inactivation, thereby potentiating its activity [21].

Several pre-clinical studies covering different disease areas have shown an increased efficacy from full-spectrum *Cannabis* extracts or combinations of CBD and THC, with or without additional compounds, compared to single compounds (e.g., THC or CBD) [4,35,36,37,38,39,40]. As an example, concerning the anti-cancer potential of *Cannabis*, Blasco-Benito et al. [36] reported a more potent anti-tumor response from a plant-based extract compared to pure THC. This enhanced therapeutic efficacy was not caused by the presence of five of the most common terpenes. It was rather believed to be a result of multiple targets and mechanisms of action affected by an array of compounds present in the extract. LaVigne et al. [41] reported that *Cannabis*-derived terpenes (i.e., α-humulene, geraniol, linalool, and β-pinene) are cannabimimetic (i.e., exerting cannabinoid-like effects) and selectively enhance the activity of cannabinoids. All terpenes activated CB1 in vitro and exerted selective additive effects when co-administered with a CB1 agonist. The authors considered these observations as evidence of the ‘entourage effect’. The terpenes β-caryophyllene and α-humulene were reported to contribute to the cytotoxic effect together with low levels of cannabichromene (CBC). CBD was, however, reported as the main cytotoxic compound in 32 different hemp inflorescences. These findings were stated to illustrate ‘inter-entourage effects’ between the terpenes and cannabinoids and ‘intra-entourage effects’ between CBC and CBD. However, antagonistic interactions from unidentified compounds in the extracts were also reported [42]. Yekhtin et al. [40] observed differential specific anti-inflammatory effects of THC- and CBD-based extract products, with a higher activity of *Cannabis* extracts compared to pure cannabinoids. However, similarities in anti-inflammatory effects exerted by the extracts with differing THC and CBD content suggest that these major cannabinoids do not solely determine the final anti-inflammatory potential related to a certain *Cannabis* extract. 

Corroborating these findings, Baram et al. [37] reported that different extracts with equivalent doses of THC affected apoptosis-inducing efficacy differently across different cancer cell lines. The fact that cancer cell lines express cannabinoid receptors differently might explain the heterogenous effects exerted by different extracts on different cell lines, even though the mechanism cannot be explained. The authors stated that matching synergistic compound compositions with specific cancer cell lines might hold the potential to optimize cancer treatment. This was supported by Li et al. [43], who observed differing anti-cancer and anti-inflammatory properties across high-THC *Cannabis* extracts from 25 different chemovars. It was concluded that the presence of certain terpenes and other cannabinoids (e.g., CBD and cannabigerol (CBG)) exerted modulating effects on THC’s anti-cancer properties. It was additionally proposed that the observed effects might be explained by the presence of compounds such as flavonoids. Namdar et al. [38] observed that only terpenes and cannabinoids co-produced in *Cannabis* chemovars exert specific interactions and induce enhanced cell cytotoxicity. This was most apparent at natural compound ratios present in *Cannabis* inflorescence (i.e., flowers)-derived extracts. These observations were concluded to be a proof of concept of the ‘inter-entourage effect’ existence. Supporting these findings, Raz et al. published a study [44] showing synergistic effects between specific terpenes and THC in CB1 activation, which was most apparent at natural ratios present in *Cannabis.* Another study [45] observed that several compounds (e.g., Δ^8^-THC, Δ^6a,10a^-THC, 11-OH-Δ^9^-THC, cannabinol (CBN), and PEA) possessed partial agonistic binding affinities at CB1 and modulated CB1 signaling in the presence of THC. 

Gallily et al. [46] found that the bell-shaped biphasic dose–response associated with the administration of purified CBD (THC Pharm. GmbH, Germany) could be overcome by the administration of a *Cannabis*-derived standardized extract highly enriched with CBD (Avidekel, i.e., clone 202, Tikun Olam, Israel). This was proposed to be caused by synergistic effects occurring between CBD and additional compounds present in the extract. It was concluded that a standardized *Cannabis* extract was recommended for the management of various inflammatory conditions. Another group of researchers [47] reported a 14-fold higher plasma concentration of cannabidiolic acid (CBDA) when administered as part of a *Cannabis* extract as opposed to as a single compound. This was explained to be the result of CBG and THC interactions with a specific drug efflux pump, causing the inhibition of the pump. As CBDA is a substrate of this pump, the inhibition results in the prevention of CBDA efflux from the blood into the intestinal lumen, which caused the enhanced plasma concentrations. It was suggested that the extract could be perceived as a natural vehicle enhancer of CBDA. 

Dahlgren et al. [48] published data derived from an open-label phase II clinical trial showing improvements in primary outcomes of anxiety-related symptoms from a full-spectrum high-CBD sublingual solution administered for four weeks. The enrolled patients (N = 14) achieved and maintained symptom reductions while experiencing few side effects. Additionally, secondary outcomes such as mood, sleep, and quality of life were observed to be improved. This was achieved by the administration of a smaller dose (approx. 30 mg/day) compared to another clinical trial testing isolated CBD from extract (approx. 300 mg/day) for its anxiolytic effects [49]. These preliminary data will be further assessed in the ongoing RCT. Similar findings have been reported by other research groups, such as Pamplona et al., who observed benefits of CBD-rich extracts in contrast to purified CBD in relation to treatment-resistant epilepsy [50]. 

Several reviews and perspectives claiming the therapeutic potential of the ‘entourage effect’ and elucidating it in a primarily optimistic perspective have been published [4,5,29,51]. These papers have in common that they refer to the same few pre-clinical original research papers presented above (e.g., [2,36,37,38]). 

### THC and CBD Combinatory Effects

Research showing beneficial therapeutical effects from co-administering THC and CBD, mainly in the form of the *Cannabis*-based medicinal product Sativex^®^ (i.e., nabiximol), has by some been interpreted as supporting evidence of the ‘entourage effect’. Sativex^®^ contains an almost equal ratio of THC and CBD, in addition to other minor compounds present in trace amounts. One heavily referenced example is the randomized controlled trial (RCT) by Johnson et al. [52] assessing the analgesic effect of Sativex^®^ compared to a THC-predominant extract and placebo in cancer patients. Sativex^®^ exerted significant analgesic effects compared to both the THC extract and placebo. As the only salient difference was the presence of CBD in Sativex^®^, it was proposed that the enhanced analgesic effect was a result of synergistic effects between THC and CBD. Sepulveda et al. [53] reported differential effects of CBD and THC regarding chemotherapy-induced neuropathic pain reduction. In general, the administration of pure THC or a high-THC extract was most effective. Pure CBD had little effect, whereas a high-CBD extract was more effective; however, it was not as potent as the high-THC extract. These differential effects might be a result of additional bioactive compounds present in the extract. 

Niesink et al. [54] performed a critical scrutiny of the scientific literature to assess the claim that CBD can protect against THC-associated adverse effects. Only a few studies supported this, however, with inconsistent results. An RCT study performed by Englund et al. [55] investigated four different THC:CBD ratios (i.e., 10 mg THC and 0, 10, 20, or 30 mg CBD) to determine if the presence of CBD in different doses improves the safety of *Cannabis* consumption. Forty-six (46) healthy infrequent *Cannabis* users reported negative outcomes from inhaling vaporized 10:0 THC:CBD mg *Cannabis*. CBD did not significantly alter this outcome at any co-administered dose. It was therefore concluded that THC:CBD co-administration in common therapeutically relevant ratios does not protect against THC-associated adverse effect acute occurrences. 

CBD’s modulation of the THC response allows for the administration of higher THC doses, potentially enhancing the clinical efficacy and creating a better safety profile. This is of particular importance to patients whose condition requires the presence of THC for therapeutic efficacy [29]. Boggs et al. [31] reviewed the pre-clinical and clinical evidence showing functional interactions between CBD and THC. The clinical data show mixed results, with some showing CBD’s potential to attenuate THC-associated adverse effects and others showing exacerbation or no impact. The authors found the scientific consensus to be limited by a scarcity of articles as well as confounding factors (e.g., self-reporting bias, variability in administration patterns, methodologies, etc.). An increasing interest in CBD’s alleviating and modulatory effects on THC’s adverse effects is evident, which has led to the development of Sativex^®^ [30]. 

The abovementioned key articles have in general been perceived as evidence supporting the ‘entourage effect’ existence, even though most of these are either pre-clinical, observational, or review articles and do not follow double-blinded randomized trials. 

## 5. Evidence Perceived as Disputing the ‘Entourage Effect’

Murataeva et al. [56] stated that different 2-AG congeners (2-LG, 2-oleoylglycerol (2-OG) and 2-PG) did not bind as orthosteric ligands to cannabinoid receptors. The congeners, however, have been suggested to potentiate the activity of 2-AG, reportedly by inhibiting its degradation. Murataeva et al. observed that the congeners did not inhibit CB1-dependent neurotransmission and failed to potentiate the 2-AG-mediated depolarization-induced suppression of excitation neuron signaling. The authors concluded that these 2-AG congeners acted as antagonists, involving neuron calcium channel inhibition and to some extent CB1 internalization. This suggests a more nuanced relationship than previously believed for ‘entourage compounds’. Another group of researchers [22] investigated the neuroprotective effects of 2-AG and PEA. When administered individually, both compounds exerted neuroprotective effects. However, when used in combination, they canceled each other’s effects. This was suggested to be caused by counteracting effects on microglial cells and possibly allosteric receptor modulation. 

Research groups [57,58] have concluded the absence of an ‘entourage effect’ involving terpenes commonly found in *Cannabis.* The terpenes did not modulate the functional activity of THC at CB1 or CB2 nor did they modulate the actions of *Cannabis*-derived cannabinoids or endocannabinoids at other non-cannabinoid receptors, i.e., transient receptor potential ankyrin 1 (TRPA1) or TRPV1 channels [59]. This might, however, differ depending on the experimental system used to assess the compounds.

In a recently published perspective paper by Cogan et al. [6], the literature perceived as supporting the ‘entourage effect’ was critically analyzed. In general, the pre-clinical and clinical studies in the field were found to be inconclusive, even contradictory, and limited in scope as they were based on only a few *Cannabis*-derived compounds.

Reviews by, among others, Russo et al. [4,5] claiming the therapeutic potential of the ‘entourage effect’ have been criticized for misinterpreting the original observations [2,3] in an attempt to scientifically back the use of full-spectrum *Cannabis* extract products with the hypothesized entourage effect term [6]. The reviews place the ‘entourage effect’ in a mostly optimistic context, with claims of almost only beneficial effects, while generally avoiding an assessment of potentially undesired adverse effects. In fact, one of these reviews [4] is among the most heavily referenced articles used to support the existence of the ‘entourage effect’. 

Cogan et al. [6] concluded that the ‘entourage effect’ term is unfounded by current research and that perspectives in favor of the ‘entourage effect’ represent a misrepresentation and abuse of the research for the benefit of marketing purposes in a currently poorly regulated medicinal *Cannabis* industry. An editorial paper titled “*Waiting for the entourage*” supported these claims [60]. The commercialization of *Cannabis*-based products has indeed grown rapidly and as a result seems to have outpaced the research field [61]. 

According to some researchers, there is no basis for expecting net beneficial effects of the ‘entourage effect’. Increased risks of adverse effects may just as well arise between the *Cannabis* compounds (e.g., cannabinoids and/or terpenes), either by augmenting negative effects or diminishing beneficial effects, which is also referred to as the ‘contra-entourage effect’ [6,7]. A study supported this [62], showing significant synergistic cytotoxic activity exerted by specific fractions of a full-spectrum *Cannabis* extract compared to the whole full-spectrum extract. Two significant effective synergistic interacting extract fractions contained the following: (1) mainly CBD (98.3%) and low concentrations of THC (0.3%), CBG (0.2%), and a trace amount of cannabidivarin (CBDV) (0.09%) and (2) mainly CBG (58.8%) and CBD (38.2%) as well as low concentrations of THC (0.7%) and CBC (0.4%), respectively. The synergistic effects were dependent on the relative ratios between the cannabinoids. These findings were proposed by the authors to not only be caused by additive effects of CBD in both fractions but also potential synergistic interactions between CBD and CBG, which has been reported by other research groups [63]. The synergy might also be a result of the activation of multiple targets and pathways besides CB1 and CB2 receptors. The depletion of non-active and antagonistic compounds from the full-spectrum extract might cause higher specific cytotoxic efficacy while reducing the concentration, and dose, of the fractions needed to exert a significant effect (i.e., a ‘contra-entourage effect’). Raup-Konsavage et al. [64] reported that CBD did not display an ‘entourage effect’ in regard to anti-cancer effects. It was observed that pure CBD exerted an equally or more potent anti-cancer effect compared to several CBD oil extracts that additionally contained minor amounts of other cannabinoids (e.g., CBC, CBG, CBN, and THC). The authors did, however, state that these in vitro observations did not rule out potential synergistic effects arising between extract compounds.

Supporting these findings, Crippa et al. [65] published data on two case studies showing worsening seizures from CBD-enriched *Cannabis*-derived extract administration in two children with treatment-refractory epilepsy. Initially, symptom improvement occurred, but it was followed by worsening symptoms, with typical signs of THC intoxication reported. Seizure remission and improvement in signs of intoxication were obtained when the children were switched to the same dose of a purified CBD product. However, these observations need to be assessed in high-quality RCTs and in the context of the administered doses. A CBD-enriched extract containing a lower THC percentage may not lead to intoxication and potentially carry therapeutic value.

In summary, when collectively assessing the data behind the proclaimed entourage effect, it is evident that the pre-clinical research primarily is based on single-compound combinations or extracts of highly unknown molecular composition. This also applies to the clinical studies in the field, where a lack of knowledge of the extract composition challenges the interpretation of the pharmacological mechanisms of action of a specific *Cannabis* extract. As illustrated in Table A1 and Table A2, the multi-targeting potential of selected *Cannabis*-derived compounds, results in complex combinatory effects, which are as of yet poorly understood [24]. 

Although evidence exists that indicates the enhanced therapeutic efficacy of extracts compared to single compounds, the question remains whether this proves the existence of the ‘entourage effect’ or if it is merely a sign of specific compound combinations exerting enhanced therapeutic efficacy (as, e.g., shown in the study by Mazuz et al. [62]). Traditional pharmacology describes this as polypharmacy, and it is the basis for active pharmaceutical ingredient (API) combinations exerting combinatory beneficial pharmacological effects. In this context, existing pharmacological terms, like additive effects, synergistic interactions, and bioenhancers, seem to be perfectly applicable in explaining the pharmacological effects underlying the proclaimed entourage effect term. For that reason, it appears unnecessary to introduce a term such as ‘entourage effect’ exclusively to *Cannabis*-based products. 

## 6. “Dirty Drugs” and Drug–Drug Interactions

In pharmacology, the informal term “dirty drug” refers to a drug that targets multiple targets within the body and exerts a wide range of effects, both desired and undesired. An example is the atypical, or second generation, antipsychotic medicinal product olanzapine, which acts as an antagonist at both dopamine (i.e., D2) and serotonin (i.e., 5-HT-2A) receptors. This exerts beneficial effects on positive as well as negative symptoms related to schizophrenic pathophysiology [66]. Olanzapine has, however, also been observed to result in adverse effects such as weight gain [67]. 

“Dirty drugs” additionally raise the risk of drug–drug interactions when co-administered with other drugs. This is particularly critical, when administering drugs with a narrow therapeutic index. Several cannabinoids are known to be metabolized by hepatic CYP enzymes, which can either inhibit or enhance other compounds’, or drugs’, metabolism and vice versa. One of the most commonly administered cannabinoids, CBD, is an inhibitor of a CYP subtype responsible for the metabolism of several antidepressants and opioids. Consequently, the hypothetical scenario of CBD co-administration with other active compounds (e.g., antidepressants or opioids) may result in an increase in API serum concentrations, ultimately affecting the resultant pharmacological response [7]. For that reason, pharmaceutical companies aim to avoid having these so-called “dirty” drugs in their pipeline and instead focus on designing drugs as selectively as possible. On the contrary, opioids and cannabinoids have been observed to exert synergistic effects in the case of, e.g., pain management, when co-administered. Thus, knowledge of specific active compound combinations exerting beneficial combinatory effects might provide alternative treatment options for patients with unmet medical needs. This might even lead to lower drug doses needed to provide a therapeutic effect, as, e.g., observed by the opioid-sparing effect of cannabinoids [68]. 

Many diseases are caused by a multifactorial causality, where single-compound medicinal products often fail to target all the disease-affected targets and as a result fail to manage the disease effectively. It has therefore been proposed that multi-compound products, such as *Cannabis* extracts, can provide beneficial effects in the management of such multifactorial diseases, being perceived as a more holistic treatment approach (e.g., [12,69]). As an example, Lehar et al. [70] observed that combinations of selected compounds resulted in synergistic effects and improved therapeutic selectivity as a result of multi-target effects. Consequently, the therapeutic dose could be reduced, additionally minimizing occurrences of adverse effects often associated with the administration of high drug doses. In fact, treatment with *Cannabis* extracts has been proposed to reduce polypharmacy, which is the realistic scenario for many patients living with multiple diseases and related symptoms. 

## 7. Factors Affecting Assessment of Potential ‘Entourage Effects’

### 7.1. Chemovar Compound Variability and Product Heterogeneity

Chemovar genotype, growth conditions, manufacturing processes, and storage conditions are all factors affecting the full-spectrum profile of a specific chemovar. The drug delivery system and route of administration of the resultant chemovar extract, or single compounds isolated from the extract, impact the final bioavailability and pharmacological effect exerted in the body [35]. The extraction method, e.g., solvent-based or supercritical CO_2_, impacts the chemical profile of the extract, highlighting the importance of improved transparency and regulation in the labeling of *Cannabis* extract products [61]. A complete profiling of the *Cannabis* product is necessary to provide proof of batch-to-batch consistency and is key to developing a truly standardized *Cannabis*-based product for medicinal purposes [71]. 

Hundreds of different *Cannabis* chemovars exist, and more than 500 compounds related to chemical classes like cannabinoids, terpenes, and flavonoids have been reported [35,72,73]. Many of these compounds possess individual bioactivities affected by the compounds they are co-administered with. This can somewhat be regarded as supporting the existence of an ‘entourage effect’, or at least combinatory effects, which, however, can be both beneficial and unfavorable. *Cannabis* has therefore been proposed to be perceived as a versatile plant and treasure trove, rather than just a single drug [35,74]. The primary focus within the research field has been on the cannabinoids THC and CBD, but these only constitute 2 out of more than 140 different cannabinoids discovered to date. Collectively, these factors contribute to the high heterogeneity of *Cannabis*-based medicinal products or derived extracts, which might partly explain the contradicting findings across studies, both pre-clinical and clinical [35]. 

### 7.2. Relative THC:CBD Ratio, Dose, and Administration Route

Across studies in the field of medicinal *Cannabis*, there are marked variabilities in dose, the route of administration, and THC:CBD ratios, in addition to relative ratios of other compounds present in different *Cannabis* extracts. These factors collectively impact pharmacological effects and clinical outcomes. Many observational studies are scarce in information on the investigated *Cannabis* product types and doses administered, which is often compared across different diseases and *Cannabis* products sourced from different suppliers. This challenges the comparison of study outcomes, resulting in questionable conclusions across reviews and meta-analyses, whenever study data are pooled [75]. 

The relative THC:CBD ratio has been observed to impact the therapeutic effect of *Cannabis*-based medicines. For example, high-THC products are administered for indications such as nausea and vomiting, whereas an equal THC:CBD ratio is beneficial in, e.g., pain and multiple sclerosis, while high-CBD products can be used for the treatment of, e.g., depression and epilepsy [76,77]. However, it is important to state that these reported effects and underlying mechanisms of actions are dose-dependent. Depending on the severity and progression of a disease, varying the dosing scheme for a specific patient is necessary. The inherent polypharmacy pertaining to multi-compound *Cannabis* extract products, in addition to the polypharmacology of the individual compounds, as illustrated in Table A1 and Table A2, will define the product’s impact on a certain pathophysiological disease state. 

The final pharmacological outcome depends on the combined effect of the co-administered compounds as they impact each other’s ability to bind to their respective targets. Furthermore, biphasic (i.e., when low and high doses exert opposite effects) dose–responses have been reported as, e.g., THC is demonstrated to be anxiolytic at low doses and anxiogenic at higher doses [78]. The biphasic effect is additionally influenced by potentially co-administered compounds. This was observed in a pre-clinical study in which the bell-shaped curve, associated with CBD administered in its pure form, was counteracted by the administration of a high-CBD extract with a full-spectrum profile [46]. A higher dose increases the risk of cannabinoid off-target binding to targets with lower affinity and selectivity [79]. THC is a partial agonist of CB1 and CB2 but can exert a mixed agonist–antagonist profile depending on the cell type and its expression of receptors, the experimental settings (e.g., in vitro or in vivo), and the co-presence of phytocannabinoids or endocannabinoids with stronger affinity to the target(s) [24,80]. 

The high lipophilicity of cannabinoids restricts their bioavailability when administered orally via conventional drug delivery strategies. Studies on ingested THC report bioavailability ranging from 4–20% [81,82], with approx. 6% reported for CBD in humans [83,84]. Many in vitro and in vivo studies in the field assess pharmacological effects in a scenario in which the compound of interest is injected directly into the target site, thus providing 100% bioavailability. This is an unrealistic pharmacological scenario in a clinical setting where the oral administration route often is preferred [79].

Thus, simple mixtures with carrier oils, like medium-chain triglycerides (MCT), being the most readily available product type on the market, lose most of their cannabinoid dose in the gastrointestinal tract following oral administration. This may partly explain the non-optimal and non-significant clinical outcomes reported across several studies in the field [35]. Therefore, more sophisticated formulations, so-called enabling technologies, are needed to increase the bioavailability and lower the dose needed to exert a therapeutic effect.

## 8. Conclusions 

Based on the published literature included within this scoping review, it is evident that there is a lack of sound evidence supporting the existence of the proclaimed *Cannabis*-related entourage effect. The literature shows contradictory, equivocal, and inconclusive findings, with both advocates and critics of the ‘entourage effect’ expressing their observations and opinions in several reviews, based primarily on the same existing original articles, of which there are relatively few. Certainly, indications exist of multi-compound and full-spectrum *Cannabis* extract products exerting enhanced efficacy and a broader therapeutic index. However, the pharmacological underlying mechanisms of action are currently based on contradictory and simplistic pre-clinical and clinical data and as such currently remain scientifically unfounded. As concluded in a recently performed review [85], focusing on potential synergistic and ‘entourage’ effects of *Cannabis*-based medicines as analgesic, further clinical trials and in vitro studies are necessary to definitively demonstrate their analgesic effects and therapeutic potential in general, both as single compounds and in various combinations. 

The ‘entourage effect’ term was originally coined as a hypothetical afterthought in a pre-clinical study to describe bio-inactive compounds potentiating a bioactive compound’s activity. The term was then used in the context of polypharmacy to refer to an enhanced therapeutic effect arising from administration of multi-compound *Cannabis*-based products, without clear justifications [6]. The ‘entourage effect’ is frequently connected to the designation of synergistic effects. This is misleading from a pharmacological point of view as it implies that the *Cannabis* compounds target the same receptor system, where an actual synergistic interaction and pharmacological amplifying effect can arise. The general reference to the ‘entourage effect’ as an enhanced therapeutic effect omits the fact that compounds present in the *Cannabis*-based product might also exert antagonistic interactions. Potentially, this can result in an unwanted adverse effect, referred to as the ‘contra-entourage effect’ [7], causing confusion when used in the context of medicinal products. Furthermore, the referral to the ‘entourage effect’ between cannabinoids and/or terpenes conflicts with the original definition of the term as most of these compounds are themselves bioactive, as illustrated in Table A1 and Table A2. 

Other natural plant-based products composed of multiple compounds have found their way into the pharmaceutical research field. Interestingly, their collective and interacting effects have not been explained as an ‘entourage effect’ but rather by the usage of traditional pharmacological terms such as synergistic/antagonistic interactions, additive effects, and bioenhancement [86]. It can therefore be speculated if the ‘entourage effect’ term is scientifically valid or if it is in fact at the borderline of *pseudoscience*. The problematic misuse of the term by the *Cannabis* industry to justify unique selling propositions of their *Cannabis* chemovars ultimately risks affecting the patients negatively. In line with Cogan et al. [6], we therefore propose that the ‘entourage effect’ is explained using established pharmacological terminology pertaining to polypharmacy in general (e.g., synergistic interactions and biohancement).

## 9. Future Perspectives

Sometimes less is more—a statement that most often applies to traditional pharmaceutical drug development, predominantly following the single-compound approach [87]. Standardized product formulations of exact compositions (i.e., APIs, e.g., cannabinoids, terpenes, and auxiliary components) with known individual and collective mechanisms of action are essential. A focus on *Cannabis*-derived ECS modulatory compounds beyond THC and CBD should be prioritized as they hold the potential for unique combinatory pharmacological effects. This should be explained by traditional pharmacological terms and verified in pre-clinical and clinical studies [23,88], resulting in less contradictory clinical outcomes, as opposed to by using “drug cocktails” with an unknown compound content and resultant unpredictable interaction effects [8,12]. A possible approach is to utilize analytical chemistry tools (e.g., high-performance liquid chromatography (HPLC)) to characterize the fingerprint of a *Cannabis* product and to understand the chemical foundation of the ‘entourage effect’. This should lead to less ambiguity in the identification of specific chemovars for certain disease treatments [89]. 

Availability of evidence-based high-quality data, in line with best practice procedures, are more likely to provide doctors with the decision basis needed to prescribe *Cannabis*-based medicine to their patients, many suffering from unmet medical needs by conventional medicinal products. Certainly, specific cannabinoid and terpene combinations hold the potential to develop design-specific *Cannabis*-based combination drugs with unique therapeutic potentials [44,90]. It has, however, been advocated that extracts can also be too pure, causing the loss of synergistic therapeutic potential [5,87]. It is required by regulatory laws to demonstrate superior clinical efficacy or the improved safety of a drug combination product composed of several APIs compared to a single API-based product [7,14,91,92]. These aspects need to be established and tackled before *Cannabis*-based products can develop into conventional medicinal products, i.e., with a solid clinical package to support doctors in their preparation of an effective treatment plan for their patients. 

## Data Availability

Not applicable.

## Appendix A

**Table A1:** Selected *Cannabis sativa* L.-derived cannabinoids, their targets, mechanisms of action, and potential resultant pharmacological effects.

Targets	Mechanisms of Action	Potential Pharmacological Effects	References
**Δ^9^-Tetrahydrocannabinol (THC)**			
CB1	Partial agonist	Analgesic **,*** Anti-convulsant ** Anti-epileptic ** Sleep improvement **,*** Anti-anorectic, appetite stimulating **,*** Anti-emetic **,*** Anxiolytic **	[27,93,94,95,96,97,98,99,100,101,102]
CB2	Partial agonist	Analgesic **,***	[94,95]
GPR55	Agonist	Not reported	[103]
GPR18	Agonist	Not reported *	[104,105]
5-HT-3A	Antagonist	Anti-nociception * Anti-emetic *	[106,107]
DOR	(Negative) Allosteric modulator	Not reported	[108,109]
MOR	(Negative) Allosteric modulator	Not reported	[108,109]
PPAR-y	Agonist	Anti-cancer, anti-proliferative *,**	[110]
GlyR	Agonist	Analgesic *,**	[111]
TRPV2	Agonist	Not reported	[32]
TRPV3	Agonist	Not reported	[32,112]
TRPV4	Agonist	Not reported	[32,112]
TRPA1	Agonist	Not reported	[113]
TRPM8	Antagonist	Not reported	[113]
**Cannabidiol (CBD)**			
CB1	Negative allosteric modulator Antagonist	THC-related adverse effects modulation **,*** Anxiolytic ** Antidepressant ** Vasorelaxant **	[27,28,95,114,115,116,117,118,119,120,121]
CB2	Partial agonist Negative allosteric modulator Antagonist	Seizure reduction ** Anti-epileptic ** Anti-inflammatory ** Anti-cancer *,** Body weight decrease ** Neuroprotection **	[27,95,114,115,122,123,124,125,126]
GPR3	Inverse agonist	Alzheimer’s disease improvement *	[127,128]
GPR6	Inverse agonist	Parkinson’s disease improvement *	[127,128]
GPR12	Inverse agonist	Anti-cancer *	[127,129]
GPR55	Antagonist	Anti-epileptic **,*** Seizure dampening ** Bone resorption inhibition ** Parkinson’s motor skills improvement ** Cancer cell migration inhibition *	[103,130,131,132,133,134]
FAAH	Inhibitor	AEA increase and related effects * Sleep induction *,** Stress reduction *** Anxiolytic *** Anti-depressant **	[135,136,137,138]
5-HT-1A	Agonist Inverse agonist	Anti-emetic *,** Analgesic ** Chemotherapy induced neuropathic pain reduction *,** Anxiolytic ** Anti-depressant ** Cognitive performance improvement ** Anti-epileptic *,**,*** Seizure reduction ** Anti-stress ** Neuroprotection **	[117,126,139,140,141,142,143,144,145,146,147,148,149,150]
5-HT-3A	Antagonist	Anti-emetic ** Cardiovascular effects **	[151,152]
A1A	Agonist	Anti-arrhythmic ** Analgesic **	[153,154]
A2A	Agonist	Anti-inflammatory *,** Cognitive performance improvement **	[155,156,157]
PPAR-γ	Agonist	β-amyloid-induced neuroinflammation reduction *,** Hippocampal neurogenesis *,** Alzheimer’s disease improvement *,**	[158]
Immune cell (not further specified)	Inhibitor Activator	Anti-inflammatory *,** Immunosuppressive *,** Cytokine release reduction/increase *,** Anti-arthritic ** Multiple sclerosis amelioration **	[159,160,161]
GlyR-α1	Positive allosteric modulator Agonist	Anti-inflammatory * Neuroprotective *	[162]
GlyR-α3	Positive allosteric modulator	Analgesic **	[163]
GABA-A	Positive allosteric modulator	Anti-convulsant ** Anti-epileptic **	[130,164]
TRPV1	Agonist	Neuron anti-hyperexcitability * Anxiolytic ** Anti-cancer, apoptosis * Microglial phagocytosis enhancement * Cardiovascular effects **	[32,135,152,165,166,167,168]
TRPV2	Agonist	Microglial phagocytosis enhancement *	[32,168]
TRPV3	Agonist	Not reported	[112]
TRPV4	Agonist	Not reported	[112]
TRPA1	Agonist	Analgesic **	[32,113,154]
TRPM8	Antagonist	Not reported	[113]
DOR	(Negative) Allosteric modulator	Not reported	[108,109]
MOR	(Negative) Allosteric modulator	Not reported	[108,109]
D2	Partial agonist	Anti-psychotic *	[169]
**Cannabigerol (CBG)**			
CB2	Partial agonist	Anti-inflammatory *,** Colitis attenuation *,**	[170,171]
AEA uptake	Inhibitor	Various effects related to AEA *	[32]
5-HT-1A	Antagonist	Reverse anti-emetic effect of, e.g., CBD **	[150,172]
A2A	Agonist	Not reported	[172]
TRPV1	Agonist	Not reported	[32]
TRPA1	Agonist	Not reported	[32,113]
TRPM8	Antagonist	Colon anti-cancer **	[32,113,173]
**Cannabichromene (CBC)**			
CB2	Agonist	Anti-inflammatory *	[174]
AEA uptake	Inhibitor	Various effects related to AEA * Analgesic **	[32,154]
TRPV3	Agonist	Not reported	[32,112]
TRPV4	Agonist	Not reported	[32,112]
TRPA1	Agonist	Anti-inflammatory ** Colitis reduction ** Analgesic **	[32,113,154,175]
TRPM8	Antagonist	Not reported	[32,113]
**Cannabinol (CBN)**			
CB1	Agonist	Appetite increase **	[176,177]
CB2	Agonist Inverse agonist	Not reported	[176,178]
TRPA1	Agonist	Not reported	[32]
TRPM8	Antagonist	Not reported	[32]
**Δ^9^-Tetrahydrocannabivarin (THCV)**			
CB1	Agonist Antagonist	Anti-psychoactive (e.g., reverse THC-induced psychoactive effects) ** Analgesic ** Anti-convulsant ** Anti-epileptic * Hypophagia and weight reduction** Glycemic control improvement**,***	[95,179,180,181,182,183,184,185]
CB2	Partial agonist Antagonist	Anti-inflammatory ** Inflammatory pain reduction **	[95,179,181]
5-HT-1A	Agonist	Antipsychotic *,**	[186]
TRPV2	Agonist	Not reported	[32]
TRPA1	Agonist	Not reported	[32]
TRPM8	Antagonist	Not reported	[32]
**Cannabidivarin (CBDV)**			
GABA-A	Positive allosteric modulator	Anti-convulsive *,*** Anti-epileptic *,***	[187]
TRPV1	Agonist	Neuronal anti-hyperexcitability * Anti-convulsant **	[32,165,188]
TRPV2	Agonist	Not reported	[32]
TRPV3	Agonist	Not reported	[32,112]
TRPA1	Agonist	Not reported	[32]
**Δ-9-Tetrahydrocannabinolic acid (THCA)**			
CB1	Partial agonist	Anti-nociceptive ** Anti-inflammatory **	[27]
CB2	Agonist	Not reported	[27]
PPAR-γ	Agonist	Adiposity reduction ** Metabolic syndrome prevention ** Anti-inflammatory ** Neuroprotective *,**	[189,190]
**Cannabidiolic acid (CBDA)**			
CB2	Partial agonist	Not reported	[27,80]
5-HT-1A	Agonist	Anti-emetic ** Anti-convulsant ** Anxiolytic **	[191,192,193]
TRPV1	Agonist	Anti-hyperalgesic **	[32,93]
**Δ^8^-Tetrahydrocannabinol (Δ^8^-THC)**			
CB1	Partial agonist	Appetite stimulant **	[194,195]
CB2	Agonist	Not reported	[194]

*: Pre-clinical in vitro study; **: pre-clinical in vivo study; ***: clinical study; N.B.: This table is non-exhaustive, broadly elucidating selected compounds and some of their potential pharmacological effects currently present in the pre-clinical literature. Depending on study parameters, the compounds show differing, sometimes biphasic, affinities and effects at different targets, thus highlighting the contradictory and equivocal evidence state. For a more extensive review on cannabinoid mechanisms of action and pharmacological effects, see these extensive reviews on the subject: Morales et al. [24], Stasiulewicz et al. [196], Almeida et al. [197], Oultram et al. [198], Vitale et al. [25], Peng et al. [199], Matheson et al. [200], Odieka et al. [71], and Castillo-Arellano et al. [26]. Abbreviations: 5-hydroxytryptamine receptor 1A (5-HT-1A); 5-hydroxytryptamine receptor 3A (5-HT-3A); adrenergic receptor alpha-1 (A1A); adrenergic receptor alpha-2 (A2A); anandamide endocannabinoid (AEA); cannabinoid receptor 1 (CB1); cannabinoid receptor 2 (CB2); delta-opioid receptor (DOR); dopamine D2 receptor (D2); fatty acid amide hydrolase enzyme (FAAH); gamma-aminobutyric acid type A receptor (GABA-A); glycine receptor (GlyR); glycine receptor type α 1 (GlyR-α1); glycine receptor type α 3 (GlyR-α3); G-protein-coupled receptor 2 (GPR2); G-protein-coupled receptor 3 (GPR3); G-protein-coupled receptor 6 (GPR6); G-protein-coupled receptor 12 (GPR12); G-protein-coupled receptor 18 (GPR18); G-protein-coupled receptor 55 (GPR55); Mu-opioid receptor (MOR); peroxisome proliferator-activated receptor gamma (PPAR-γ); transient receptor potential cation channel type A1 (TRPA1); transient receptor potential cation channel 8 (TRPM8); transient receptor potential vanilloid type 1 (TRPV1); transient receptor potential vanilloid type 2 (TRPV2); transient receptor potential vanilloid type 3 (TRPV3); transient receptor potential vanilloid type 4 (TRPV4).

## Appendix B

**Table A2:** Selected *Cannabis sativa* L.-derived terpenes, their targets, mechanisms of action, and potential resultant pharmacological effects.

Targets	Mechanisms of Action	Potential Pharmacological Effects	References
**Caryophyllene**			
CB2	Agonist	Analgesic ** Chemotherapy-induced peripheral neuropathy attenuation ** Anti-inflammatory ** Steatohepatitis protecting ** Metabolic dysregulation attenuation **	[201,202,203,204,205,206,207]
PPAR-α	Agonist	Intracellular lipid modification * Steatohepatitis protecting *	[207]
PPAR-γ	Agonist	Intracellular lipid modification * Steatohepatitis protecting *	[207]
MAPK	Inhibitor Agonist	Chemotherapy-induced peripheral neuropathy attenuation ** Anti-cancer *	[206,208]
TLR4	Inhibitor	Microglial activation inhibition ** Neuroprotective *,** Anti-inflammatory *,**	[209,210]
**Limonene**			
5-HT-1A	Agonist	Anti-stress ** Anxiolytic ** Anti-depressant **	[211]
TRPA1	Agonist	Analgesic **	[212]
NFκB	Inhibitor	Anti-inflammatory **,*** Analgesic ** Colitis reduction **	[213,214]
A2A	Agonist	Not reported	[215]
FTase	Inhibitor	Anti-cancer ***	[216]
**Pinene**			
MAPK NFκB	Inhibitor	Anti-inflammatory **	[217]
ERK/AKT	Agonist	Anti-cancer *,**	[218]
Virus particle (not further specified)	Inhibitor	Anti-viral *	[219]
**Myrcene**			
TRPV1	Agonist	Analgesic *	[220]
A2A	Agonist	Analgesic **	[221]
**Linalool**			
A1A	Agonist	Analgesic **	[222]
A2A	Agonist	Analgesic **	[222]
GABA-A	Agonist	Anxiolytic **	[223]
Cancer cell (not further specified)	Inhibitor	Anti-cancer *,**	[224]

* Pre-clinical in vitro study. ** Pre-clinical in vivo study. *** Clinical study. N.B.: This table is non-exhaustive, broadly elucidating selected compounds and some of their potential pharmacological effects currently present in the pre-clinical literature. Depending on study parameters, the compounds show differing, sometimes biphasic, affinities and effects at different targets, thus highlighting the contradictory and equivocal evidence state. For a more extensive review on terpene mechanisms of actions and pharmacological effects, see these extensive reviews on the subject: Goncalves et al. [225], Liktor-Busa et al. [226], and Odieka et al. [33,71]. Abbreviations: 5-hydroxytryptamine receptor 1A (5-HT-1A); adrenergic receptor alpha-1 (A1A); adrenergic receptor alpha-2 (A2A); cannabinoid receptor 2 (CB2); Extracellular-regulated kinase/serine/threonine kinase (ERK/AKT); Farnesyltransferase (FTase); gamma-aminobutyric acid type A receptor(GABA-A); mitogen-activated protein kinase (MAPK); Nuclear factor kappa B (NFκB); peroxisome proliferator-activated receptor alpha/gamma (PPAR-α/γ); Toll-like receptor 4 (TLR4); transient receptor potential cation channel type A1 (TRPA1); transient receptor potential vanilloid type 1 (TRPV1).

## References

[1] Lowe H., Toyang N., Steele B., Bryant J., Ngwa W. (2021). The Endocannabinoid System: A Potential Target for the Treatment of Various Diseases. Int. J. Mol. Sci..

[2] Ben-Shabat S., Fride E., Sheskin T., Tamiri T., Rhee M.-H., Vogel Z., Bisogno T., De Petrocellis L., Marzo V.D., Mechoulam R. (1998). An Entourage Effect: Inactive Endogenous Fatty Acid Glycerol Esters Enhance 2-Arachidonoyl-Glycerol Cannabinoid Activity. Eur. J. Pharmacol..

[3] Mechoulam R., Ben-Shabat S. (1999). From Gan-Zi-Gun-Nu to Anandamide and 2-Arachidonoylglycerol: The Ongoing Story of Cannabis. Nat. Prod. Rep..

[4] Russo E.B. (2011). Taming THC: Potential Cannabis Synergy and Phytocannabinoid-Terpenoid Entourage Effects. Br. J. Pharmacol..

[5] Russo E.B. (2019). The Case for the Entourage Effect and Conventional Breeding of Clinical Cannabis: No “Strain”, No Gain. Front. Plant Sci..

[6] Cogan P.S. (2020). The ‘Entourage Effect’ or ‘Hodge-Podge Hashish’: The Questionable Rebranding, Marketing, and Expectations of Cannabis Polypharmacy. Expert. Rev. Clin. Pharmacol..

[7] Namdar D., Anis O., Poulin P., Koltai H. (2020). Chronological Review and Rational and Future Prospects of Cannabis-Based Drug Development. Molecules.

[8] Worth T. (2019). Unpicking the Entourage Effect. Nature.

[9] McPartland J.M., Russo E.B. (2014). Synergy, Additivity, and Antagonism. Handbook of Cannabis.

[10] Yang Y., Zhang Z., Li S., Ye X., Li X., He K. (2014). Synergy Effects of Herb Extracts: Pharmacokinetics and Pharmacodynamic Basis. Fitoterapia.

[11] Niu J., Straubinger R.M., Mager D.E. (2019). Pharmacodynamic Drug–Drug Interactions. Clin. Pharmacol. Ther..

[12] Caesar L.K., Cech N.B. (2019). Synergy and Antagonism in Natural Product Extracts: When 1 + 1 Does Not Equal 2. Nat. Prod. Rep..

[13] Roell K.R., Reif D.M., Motsinger-Reif A.A. (2017). An Introduction to Terminology and Methodology of Chemical Synergy-Perspectives from across Disciplines. Front. Pharmacol..

[14] Wagner H. (2011). Synergy Research: Approaching a New Generation of Phytopharmaceuticals. Fitoterapia.

[15] Zafar N., Pharm M. (2017). Herbal Bioenhancers: A Revolutionary Concept in Modern Medicine. Zafar World J. Pharm. Res..

[16] Lucas C.J., Galettis P., Schneider J. (2018). The Pharmacokinetics and the Pharmacodynamics of Cannabinoids. Br. J. Clin. Pharmacol..

[17] Peterson B., Weyers M., Steenekamp J.H., Steyn J.D., Gouws C., Hamman J.H. (2019). Drug Bioavailability Enhancing Agents of Natural Origin (Bioenhancers) That Modulate Drug Membrane Permeation and Pre-Systemic Metabolism. Pharmaceutics.

[18] Kulkarni D., Surwase S., Musale S., Giram P. (2022). Current Trends on Herbal Bioenhancers. Drug Delivery Technology: Herbal Bioenhancers in Pharmaceuticals.

[19] Anand U., Pacchetti B., Anand P., Sodergren M.H. (2022). The Endocannabinoid Analgesic Entourage Effect: Investigations in Cultured DRG Neurons. J. Pain. Res..

[20] Ho W.-S., Barrett D.A., Randall M.D. (2008). ‘Entourage’ Effects of N-Palmitoylethanolamide and N-Oleoylethanolamide on Vasorelaxation to Anandamide Occur through TRPV1 Receptors. Br. J. Pharmacol..

[21] Jonsson K.-O., Vandevoorde S., Lambert D.M., Tiger G., Fowler C.J. (2001). Effects of Homologues and Analogues of Palmitoylethanolamide upon the Inactivation of the Endocannabinoid Anandamide. Br. J. Pharmacol..

[22] Hohmann U., Pelzer M., Kleine J., Hohmann T., Ghadban C., Dehghani F. (2019). Opposite Effects of Neuroprotective Cannabinoids, Palmitoylethanolamide, and 2-Arachidonoylglycerol on Function and Morphology of Microglia. Front. Neurosci..

[23] Koltai H., Namdar D. (2020). Cannabis Phytomolecule “Entourage”: From Domestication to Medical Use. Trends Plant Sci..

[24] Morales P., Hurst D.P., Reggio P.H. (2017). Molecular Targets of the Phytocannabinoids: A Complex Picture. Prog. Chem. Org. Nat. Prod..

[25] Vitale R.M., Iannotti F.A., Amodeo P. (2021). The (Poly)Pharmacology of Cannabidiol in Neurological and Neuropsychiatric Disorders: Molecular Mechanisms and Targets. Int. J. Mol. Sci..

[26] Castillo-Arellano J., Canseco-Alba A., Cutler S.J., León F. (2023). The Polypharmacological Effects of Cannabidiol. Molecules.

[27] Zagzoog A., Mohamed K.A., Kim H.J.J., Kim E.D., Frank C.S., Black T., Jadhav P.D., Holbrook L.A., Laprairie R.B. (2020). In Vitro and In Vivo Pharmacological Activity of Minor Cannabinoids Isolated from *Cannabis Sativa*. Sci. Rep..

[28] Laprairie R.B., Bagher A.M., Kelly M.E.M., Denovan-Wright E.M. (2015). Cannabidiol Is a Negative Allosteric Modulator of the Cannabinoid CB1 Receptor. Br. J. Pharmacol..

[29] Russo E., Guy G.W. (2006). A Tale of Two Cannabinoids: The Therapeutic Rationale for Combining Tetrahydrocannabinol and Cannabidiol. Med. Hypotheses.

[30] McPartland J.M., Russo E.B. (2014). Modern Synergy Research. Handbook of Cannabis.

[31] Boggs D.L., Nguyen J.D., Morgenson D., Taffe M.A., Ranganathan M. (2018). Clinical and Preclinical Evidence for Functional Interactions of Cannabidiol and Δ9-Tetrahydrocannabinol. Neuropsychopharmacology.

[32] De Petrocellis L., Ligresti A., Schiano Moriello A., Allarà M., Bisogno T., Petrosino S., Stott C.G., Marzo V.D. (2011). Effects of Cannabinoids and Cannabinoid-Enriched Cannabis Extracts on TRP Channels and Endocannabinoid Metabolic Enzymes. Br. J. Pharmacol..

[33] Chacon F.T., Raup-Konsavage W.M., Vrana K.E., Kellogg J.J. (2022). Secondary Terpenes in *Cannabis sativa* L.: Synthesis and Synergy. Biomedicines.

[34] Bautista J.L., Yu S., Tian L. (2021). Flavonoids in *Cannabis Sativa*: Biosynthesis, Bioactivities, and Biotechnology. ACS Omega.

[35] Procaccia S., Lewitus G.M., Lipson Feder C., Shapira A., Berman P., Meiri D. (2022). Cannabis for Medical Use: Versatile Plant Rather Than a Single Drug. Front. Pharmacol..

[36] Blasco-Benito S., Seijo-Vila M., Caro-Villalobos M., Tundidor I., Andradas C., García-Taboada E., Wade J., Smith S., Guzmán M., Pérez-Gómez E. (2018). Appraising the “Entourage Effect”: Antitumor Action of a Pure Cannabinoid versus a Botanical Drug Preparation in Preclinical Models of Breast Cancer. Biochem. Pharmacol..

[37] Baram L., Peled E., Berman P., Yellin B., Besser E., Benami M., Louria-Hayon I., Lewitus G.M., Meiri D. (2019). The Heterogeneity and Complexity of Cannabis Extracts as Antitumor Agents. Oncotarget.

[38] Namdar D., Voet H., Ajjampura V., Nadarajan S., Mayzlish-Gati E., Mazuz M., Shalev N., Koltai H. (2019). Terpenoids and Phytocannabinoids Co-Produced in *Cannabis Sativa* Strains Show Specific Interaction for Cell Cytotoxic Activity. Molecules.

[39] Ferber S.G., Namdar D., Hen-Shoval D., Eger G., Koltai H., Shoval G., Shbiro L., Weller A. (2019). The “Entourage Effect”: Terpenes Coupled with Cannabinoids for the Treatment of Mood Disorders and Anxiety Disorders. Curr. Neuropharmacol..

[40] Yekhtin Z., Khuja I., Meiri D., Or R., Almogi-Hazan O. (2022). Differential Effects of D9 Tetrahydrocannabinol (THC)- and Cannabidiol (CBD)-Based Cannabinoid Treatments on Macrophage Immune Function In Vitro and on Gastrointestinal Inflammation in a Murine Model. Biomedicines.

[41] LaVigne J.E., Hecksel R., Keresztes A., Streicher J.M. (2021). *Cannabis Sativa* Terpenes Are Cannabimimetic and Selectively Enhance Cannabinoid Activity. Sci. Rep..

[42] Di Giacomo S., Mariano A., Gullì M., Fraschetti C., Vitalone A., Filippi A., Mannina L., Scotto D’Abusco A., Di Sotto A. (2021). Role of Caryophyllane Sesquiterpenes in the Entourage Effect of Felina 32 Hemp Inflorescence Phytocomplex in Triple Negative MDA-MB-468 Breast Cancer Cells. Molecules.

[43] Li D., Ilnytskyy Y., Ghasemi Gojani E., Kovalchuk O., Kovalchuk I. (2022). Analysis of Anti-Cancer and Anti-Inflammatory Properties of 25 High-THC Cannabis Extracts. Molecules.

[44] Raz N., Eyal A.M., Zeitouni D.B., Hen-Shoval D., Davidson E.M., Danieli A., Tauber M., Ben-Chaim Y. (2023). Selected Cannabis Terpenes Synergize with THC to Produce Increased CB1 Receptor Activation. Biochem. Pharmacol..

[45] Zagzoog A., Cabecinha A., Abramovici H., Laprairie R.B. (2022). Modulation of Type 1 Cannabinoid Receptor Activity by Cannabinoid By-Products from *Cannabis Sativa* and Non-Cannabis Phytomolecules. Front. Pharmacol..

[46] Gallily R., Yekhtin Z., Hanuš L.O. (2015). Overcoming the Bell-Shaped Dose-Response of Cannabidiol by Using Cannabis Extract Enriched in Cannabidiol. Pharmacol. Pharm..

[47] Anderson L.L., Etchart M.G., Bahceci D., Golembiewski T.A., Arnold J.C. (2021). Cannabis Constituents Interact at the Drug Efflux Pump BCRP to Markedly Increase Plasma Cannabidiolic Acid Concentrations. Sci. Rep..

[48] Dahlgren M.K., Lambros A.M., Smith R.T., Sagar K.A., El-Abboud C., Gruber S.A. (2022). Clinical and Cognitive Improvement Following Full-Spectrum, High-Cannabidiol Treatment for Anxiety: Open-Label Data from a Two-Stage, Phase 2 Clinical Trial. Commun. Med..

[49] Masataka N. (2019). Anxiolytic Effects of Repeated Cannabidiol Treatment in Teenagers with Social Anxiety Disorders. Front. Psychol..

[50] Pamplona F.A., Da Silva L.R., Coan A.C. (2018). Potential Clinical Benefits of CBD-Rich Cannabis Extracts over Purified CBD in Treatment-Resistant Epilepsy: Observational Data Meta-Analysis. Front. Neurol..

[51] McPartland J.M., Russo E.B. (2001). Cannabis and Cannabis Extracts: Greater Than the Sum of Their Parts?. J. Cannabis Ther..

[52] Johnson J.R., Burnell-Nugent M., Lossignol D., Ganae-Motan E.D., Potts R., Fallon M.T. (2010). Multicenter, Double-Blind, Randomized, Placebo-Controlled, Parallel-Group Study of the Efficacy, Safety, and Tolerability of THC:CBD Extract and THC Extract in Patients with Intractable Cancer-Related Pain. J. Pain. Symptom Manag..

[53] Sepulveda D.E., Vrana K.E., Graziane N.M., Raup-Konsavage W.M. (2022). Combinations of Cannabidiol and Δ9-Tetrahydrocannabinol in Reducing Chemotherapeutic Induced Neuropathic Pain. Biomedicines.

[54] Niesink R.J.M., van Laar M.W. (2013). Does Cannabidiol Protect Against Adverse Psychological Effects of THC?. Front. Psychiatry.

[55] Englund A., Oliver D., Chesney E., Chester L., Wilson J., Sovi S., De Micheli A., Hodsoll J., Fusar-Poli P., Strang J. (2022). Does Cannabidiol Make Cannabis Safer? A Randomised, Double-Blind, Cross-over Trial of Cannabis with Four Different CBD:THC Ratios. Neuropsychopharmacology.

[56] Murataeva N., Dhopeshwarkar A., Yin D., Mitjavila J., Bradshaw H., Straiker A., MacKie K. (2016). Where’s My Entourage? The Curious Case of 2-Oleoylglycerol, 2-Linolenoylglycerol, and 2-Palmitoylglycerol. Pharmacol. Res..

[57] Santiago M., Sachdev S., Arnold J.C., Mcgregor I.S., Connor M. (2019). Absence of Entourage: Terpenoids Commonly Found in *Cannabis Sativa* Do Not Modulate the Functional Activity of Δ9-THC at Human CB1 and CB2 Receptors. Cannabis Cannabinoid Res..

[58] Finlay D.B., Sircombe K.J., Nimick M., Jones C., Glass M. (2020). Terpenoids from Cannabis Do Not Mediate an Entourage Effect by Acting at Cannabinoid Receptors. Front. Pharmacol..

[59] Heblinski M., Santiago M., Fletcher C., Stuart J., Connor M., McGregor I.S., Arnold J.C. (2020). Terpenoids Commonly Found in *Cannabis Sativa* Do Not Modulate the Actions of Phytocannabinoids or Endocannabinoids on TRPA1 and TRPV1 Channels. Cannabis Cannabinoid Res..

[60] Piomelli D. (2019). Waiting for the Entourage. Cannabis Cannabinoid Res..

[61] Bowen J.K., Chaparro J.M., McCorkle A.M., Palumbo E., Prenni J.E. (2021). The Impact of Extraction Protocol on the Chemical Profile of Cannabis Extracts from a Single Cultivar. Sci. Rep..

[62] Mazuz M., Tiroler A., Moyal L., Hodak E., Nadarajan S., Vinayaka A.C., Gorovitz-Haris B., Lubin I., Drori A., Drori G. (2020). Synergistic Cytotoxic Activity of Cannabinoids from *Cannabis Sativa* against Cutaneous T-Cell Lymphoma (CTCL) In Vitro and Ex Vivo. Oncotarget.

[63] Scott K.A., Shah S., Dalgleish A.G., Liu W.M. (2013). Enhancing the Activity of Cannabidiol and Other Cannabinoids In Vitro through Modifications to Drug Combinations and Treatment Schedules. Anticancer. Res..

[64] Raup-Konsavage W.M., Carkaci-Salli N., Greenland K., Gearhart R., Vrana K.E. (2020). Cannabidiol (CBD) Oil Does Not Display an Entourage Effect in Reducing Cancer Cell Viability In Vitro. Med. Cannabis Cannabinoids.

[65] Crippa J.A.S., Crippa A.C.S., Hallak J.E.C., Martín-Santos R., Zuardi A.W. (2016). Δ9-THC Intoxication by Cannabidiol-Enriched Cannabis Extract in Two Children with Refractory Epilepsy: Full Remission after Switching to Purified Cannabidiol. Front. Pharmacol..

[66] Thomas K., Saadabadi A. (2023). Olanzapine.

[67] Hou P.H., Chang G.R., Chen C.P., Lin Y.L., Chao I.S., Shen T.T., Mao F.C. (2018). Long-Term Administration of Olanzapine Induces Adiposity and Increases Hepatic Fatty Acid Desaturation Protein in Female C57bl/6j Mice. Iran. J. Basic. Med. Sci..

[68] Nielsen S., Picco L., Murnion B., Winters B., Matheson J., Graham M., Campbell G., Parvaresh L., Khor K.E., Betz-Stablein B. (2022). Opioid-Sparing Effect of Cannabinoids for Analgesia: An Updated Systematic Review and Meta-Analysis of Preclinical and Clinical Studies. Neuropsychopharmacology.

[69] Frantz S. (2005). Playing Dirty. Nature.

[70] Lehár J., Krueger A.S., Avery W., Heilbut A.M., Johansen L.M., Price E.R., Rickles R.J., Short G.F., Staunton J.E., Jin X. (2009). Synergistic Drug Combinations Tend to Improve Therapeutically Relevant Selectivity. Nat. Biotechnol..

[71] Odieka A.E., Obuzor G.U., Oyedeji O.O., Gondwe M., Hosu Y.S., Oyedeji A.O. (2022). The Medicinal Natural Products of *Cannabis Sativa* Linn.: A Review. Molecules.

[72] Lowe H., Steele B., Bryant J., Toyang N., Ngwa W. (2021). Non-Cannabinoid Metabolites of *Cannabis Sativa* L. with Therapeutic Potential. Plants.

[73] Radwan M.M., Chandra S., Gul S., Elsohly M.A. (2021). Cannabinoids, Phenolics, Terpenes and Alkaloids of Cannabis. Molecules.

[74] Owens B. (2015). Drug Development: The Treasure Chest. Nature.

[75] Jugl S., Sajdeya R., Morris E.J., Goodin A.J., Brown J.D. (2021). Much Ado about Dosing: The Needs and Challenges of Defining a Standardized Cannabis Unit. Med. Cannabis Cannabinoids.

[76] Brunetti P., Pichini S., Pacifici R., Busardò F.P., del Rio A. (2020). Herbal Preparations of Medical Cannabis: A Vademecum for Prescribing Doctors. Medicina.

[77] Nahler G. (2019). Cannabidiol and Contributions of Major Hemp Phytocompounds to the “Entourage Effect”; Possible Mechanisms. Altern. Complement. Integr. Med..

[78] Katsidoni V., Kastellakis A., Panagis G. (2013). Biphasic Effects of Δ9-Tetrahydrocannabinol on Brain Stimulation Reward and Motor Activity. Int. J. Neuropsychopharmacol..

[79] Franco R., Rivas-Santisteban R., Reyes-Resina I., Casanovas M., Pérez-Olives C., Ferreiro-Vera C., Navarro G., Sánchez de Medina V., Nadal X. (2020). Pharmacological Potential of Varinic-, Minor-, and Acidic Phytocannabinoids. Pharmacol. Res..

[80] Navarro G., Varani K., Lillo A., Vincenzi F., Rivas-Santisteban R., Raïch I., Reyes-Resina I., Ferreiro-Vera C., Borea P.A., Sánchez de Medina V. (2020). Pharmacological Data of Cannabidiol- and Cannabigerol-Type Phytocannabinoids Acting on Cannabinoid CB1, CB2 and CB1/CB2 Heteromer Receptors. Pharmacol. Res..

[81] Wall M.E., Sadler B.M., Brine D., Taylor H., Perez-Reyes M. (1983). Metabolism, Disposition, and Kinetics of Delta-9-Tetrahydrocannabinol in Men and Women. Clin. Pharmacol. Ther..

[82] McGilveray I.J. (2005). Pharmacokinetics of Cannabinoids. Pain. Res. Manag..

[83] Chayasirisobhon S. (2020). Mechanisms of Action and Pharmacokinetics of Cannabis. Perm. J..

[84] Devinsky O., Cilio M.R., Cross H., Fernandez-Ruiz J., French J., Hill C., Katz R., Di Marzo V., Jutras-Aswad D., Notcutt W.G. (2014). Cannabidiol: Pharmacology and Potential Therapeutic Role in Epilepsy and Other Neuropsychiatric Disorders. Epilepsia.

[85] Anand U., Pacchetti B., Anand P., Sodergren M.H. (2021). Cannabis-Based Medicines and Pain: A Review of Potential Synergistic and Entourage Effects. Pain Manag..

[86] Rajčević N., Bukvički D., Dodoš T., Marin P.D. (2022). Interactions between Natural Products—A Review. Metabolites.

[87] Bonn-Miller M.O., ElSohly M.A., Loflin M.J.E., Chandra S., Vandrey R. (2018). Cannabis and Cannabinoid Drug Development: Evaluating Botanical versus Single Molecule Approaches. Int. Rev. Psychiatry.

[88] Koltai H., Poulin P., Namdar D. (2019). Promoting Cannabis Products to Pharmaceutical Drugs. Eur. J. Pharm. Sci..

[89] Silva Sofrás F.M., Desimone M.F. (2023). Entourage Effect and Analytical Chemistry: Chromatography as a Tool in the Analysis of the Secondary Metabolism of *Cannabis sativa* L.. Curr. Pharm. Des..

[90] Raz N., Eyal A.M., Davidson E.M. (2022). Optimal Treatment with Cannabis Extracts Formulations Is Gained via Knowledge of Their Terpene Content and via Enrichment with Specifically Selected Monoterpenes and Monoterpenoids. Molecules.

[91] U.S. Food and Drug Administration (FDA) Guidance for Industry and FDA Staff: Current Good Manufacturing Practice Requirements for Combination Products. https://www.fda.gov/regulatory-information/search-fda-guidance-documents/current-good-manufacturing-practice-requirements-combination-products.

[92] U.S. Food and Drug Administration (FDA) FDA Regulation of Cannabis and Cannabis-Derived Products, Including Cannabidiol (CBD). https://www.fda.gov/news-events/public-health-focus/fda-regulation-cannabis-and-cannabis-derived-products-including-cannabidiol-cbd.

[93] Rock E.M., Limebeer C.L., Parker L.A. (2018). Effect of Cannabidiolic Acid and ∆9-Tetrahydrocannabinol on Carrageenan-Induced Hyperalgesia and Edema in a Rodent Model of Inflammatory Pain. Psychopharmacology.

[94] Rahn E.J., Hohmann A.G. (2009). Cannabinoids as Pharmacotherapies for Neuropathic Pain: From the Bench to the Bedside. Neurotherapeutics.

[95] Pertwee R.G. (2008). The Diverse CB_1_ and CB_2_ Receptor Pharmacology of Three Plant Cannabinoids: Δ^9^-Tetrahydrocannabinol, Cannabidiol and Δ^9^-Tetrahydrocannabivarin. Br. J. Pharmacol..

[96] Brafford May M., Glode A. (2016). Dronabinol for Chemotherapy-Induced Nausea and Vomiting Unresponsive to Antiemetics. Cancer Manag. Res..

[97] Darmani N.A., Chebolu S., Zhong W., Trinh C., McClanahan B., Brar R.S. (2014). Additive Antiemetic Efficacy of Low-Doses of the Cannabinoid CB_1/2_ Receptor Agonist Δ^9^-THC with Ultralow-Doses of the Vanilloid TRPV1 Receptor Agonist Resiniferatoxin in the Least Shrew (Cryptotis Parva). Eur. J. Pharmacol..

[98] Beal J.E., Olson R., Laubenstein L., Morales J.O., Bellman P., Yangco B., Lefkowitz L., Plasse T.F., Shepard K. (1995). Dronabinol as a Treatment for Anorexia Associated with Weight Loss in Patients with AIDS. J. Pain Symptom Manag..

[99] Wallace M.J., Blair R.E., Falenski K.W., Martin B.R., DeLorenzo R.J. (2003). The Endogenous Cannabinoid System Regulates Seizure Frequency and Duration in a Model of Temporal Lobe Epilepsy. J. Pharmacol. Exp. Ther..

[100] Murillo-Rodríguez E. (2008). The Role of the CB1 Receptor in the Regulation of Sleep. Prog. Neuropsychopharmacol. Biol. Psychiatry.

[101] Darmani N.A., Belkacemi L., Zhong W. (2019). Δ^9^-THC and Related Cannabinoids Suppress Substance P-Induced Neurokinin NK1-Receptor-Mediated Vomiting via Activation of Cannabinoid CB1 Receptor. Eur. J. Pharmacol..

[102] Rubino T., Sala M., Viganò D., Braida D., Castiglioni C., Limonta V., Guidali C., Realini N., Parolaro D. (2007). Cellular Mechanisms Underlying the Anxiolytic Effect of Low Doses of Peripheral Δ9-Tetrahydrocannabinol in Rats. Neuropsychopharmacology.

[103] Ryberg E., Larsson N., Sjögren S., Hjorth S., Hermansson N.-O., Leonova J., Elebring T., Nilsson K., Drmota T., Greasley P.J. (2007). The Orphan Receptor GPR55 Is a Novel Cannabinoid Receptor. Br. J. Pharmacol..

[104] McHugh D., Page J., Dunn E., Bradshaw H.B. (2012). Δ9-Tetrahydrocannabinol and N-Arachidonyl Glycine Are Full Agonists at GPR18 Receptors and Induce Migration in Human Endometrial HEC-1B Cells. Br. J. Pharmacol..

[105] Kotańska M., Kubacka M., Bednarski M., Nicosia N., Szafarz M., Jawień W., Müller C.E., Kieć-Kononowicz K. (2021). The GPR18 Agonist PSB-KD-107 Exerts Endothelium-Dependent Vasorelaxant Effects. Pharmaceuticals.

[106] Barann M., Molderings G., Brüss M., Bönisch H., Urban B.W., Göthert M. (2002). Direct Inhibition by Cannabinoids of Human 5-HT3A Receptors: Probable Involvement of an Allosteric Modulatory Site. Br. J. Pharmacol..

[107] Yang K.H.S., Isaev D., Morales M., Petroianu G., Galadari S., Oz M. (2010). The Effect of Δ9-Tetrahydrocannabinol on 5-HT3 Receptors Depends on the Current Density. Neuroscience.

[108] Kathmann M., Flau K., Redmer A., Tränkle C., Schlicker E. (2006). Cannabidiol Is an Allosteric Modulator at Mu- and Delta-Opioid Receptors. Naunyn Schmiedebergs Arch. Pharmacol..

[109] Livingston K.E., Traynor J.R. (2018). Allostery at Opioid Receptors: Modulation with Small Molecule Ligands. Br. J. Pharmacol..

[110] Vara D., Morell C., Rodríguez-Henche N., Diaz-Laviada I. (2013). Involvement of PPARγ in the Antitumoral Action of Cannabinoids on Hepatocellular Carcinoma. Cell Death Dis..

[111] Xiong W., Cheng K., Cui T., Godlewski G., Rice K.C., Xu Y., Zhang L. (2011). Cannabinoid Potentiation of Glycine Receptors Contributes to Cannabis-Induced Analgesia. Nat. Chem. Biol..

[112] De Petrocellis L., Orlando P., Moriello A.S., Aviello G., Stott C., Izzo A.A., Di Marzo V. (2012). Cannabinoid Actions at TRPV Channels: Effects on TRPV3 and TRPV4 and Their Potential Relevance to Gastrointestinal Inflammation. Acta Physiol..

[113] De Petrocellis L., Vellani V., Schiano-Moriello A., Marini P., Magherini P.C., Orlando P., Di Marzo V. (2008). Plant-Derived Cannabinoids Modulate the Activity of Transient Receptor Potential Channels of Ankyrin Type-1 and Melastatin Type-8. J. Pharmacol. Exp. Ther..

[114] Tham M., Yilmaz O., Alaverdashvili M., Kelly M.E.M., Denovan-Wright E.M., Laprairie R.B. (2019). Allosteric and Orthosteric Pharmacology of Cannabidiol and Cannabidiol-Dimethylheptyl at the Type 1 and Type 2 Cannabinoid Receptors. Br. J. Pharmacol..

[115] Thomas A., Baillie G.L., Phillips A.M., Razdan R.K., Ross R.A., Pertwee R.G. (2007). Cannabidiol Displays Unexpectedly High Potency as an Antagonist of CB1 and CB2 Receptor Agonists in Vitro. Br. J. Pharmacol..

[116] Morales P., Goya P., Jagerovic N., Hernandez-Folgado L. (2016). Allosteric Modulators of the CB1 Cannabinoid Receptor: A Structural Update Review. Cannabis Cannabinoid Res..

[117] Sartim A.G., Guimarães F.S., Joca S.R.L. (2016). Antidepressant-like Effect of Cannabidiol Injection into the Ventral Medial Prefrontal Cortex—Possible Involvement of 5-HT1A and CB1 Receptors. Behav. Brain Res..

[118] Austrich-Olivares A., García-Gutiérrez M.S., Illescas L., Gasparyan A., Manzanares J. (2022). Cannabinoid CB1 Receptor Involvement in the Actions of CBD on Anxiety and Coping Behaviors in Mice. Pharmaceuticals.

[119] Stanley C.P., Hind W.H., Tufarelli C., O’Sullivan S.E. (2015). Cannabidiol Causes Endothelium-Dependent Vasorelaxation of Human Mesenteric Arteries via CB1 Activation. Cardiovasc. Res..

[120] Morgan C.J.A., Schafer G., Freeman T.P., Curran H.V. (2010). Impact of Cannabidiol on the Acute Memory and Psychotomimetic Effects of Smoked Cannabis: Naturalistic Study. Br. J. Psychiatry.

[121] Hayakawa K., Mishima K., Hazekawa M., Sano K., Irie K., Orito K., Egawa T., Kitamura Y., Uchida N., Nishimura R. (2008). Cannabidiol Potentiates Pharmacological Effects of Δ9-Tetrahydrocannabinol via CB1 Receptor-Dependent Mechanism. Brain Res..

[122] Martínez-Pinilla E., Varani K., Reyes-Resina I., Angelats E., Vincenzi F., Ferreiro-Vera C., Oyarzabal J., Canela E.I., Lanciego J.L., Nadal X. (2017). Binding and Signaling Studies Disclose a Potential Allosteric Site for Cannabidiol in Cannabinoid CB2 Receptors. Front. Pharmacol..

[123] Ligresti A., Moriello A.S., Starowicz K., Matias I., Pisanti S., De Petrocellis L., Laezza C., Portella G., Bifulco M., Di Marzo V. (2006). Antitumor Activity of Plant Cannabinoids with Emphasis on the Effect of Cannabidiol on Human Breast Carcinoma. J. Pharmacol. Exp. Ther..

[124] Ji X., Zeng Y., Wu J. (2021). The CB2 Receptor as a Novel Therapeutic Target for Epilepsy Treatment. Int. J. Mol. Sci..

[125] Ignatowska-Jankowska B., Jankowski M.M., Swiergiel A.H. (2011). Cannabidiol Decreases Body Weight Gain in Rats: Involvement of CB2 Receptors. Neurosci. Lett..

[126] Pazos M.R., Mohammed N., Lafuente H., Santos M., Martínez-Pinilla E., Moreno E., Valdizan E., Romero J., Pazos A., Franco R. (2013). Mechanisms of Cannabidiol Neuroprotection in Hypoxic–Ischemic Newborn Pigs: Role of 5HT1A and CB2 Receptors. Neuropharmacology.

[127] Laun A.S., Shrader S.H., Brown K.J., Song Z.H. (2019). GPR3, GPR6, and GPR12 as Novel Molecular Targets: Their Biological Functions and Interaction with Cannabidiol. Acta Pharmacol. Sin..

[128] Laun A.S., Song Z.-H. (2017). GPR3 and GPR6, Novel Molecular Targets for Cannabidiol. Biochem. Biophys. Res. Commun..

[129] Brown K.J., Laun A.S., Song Z.-H. (2017). Cannabidiol, a Novel Inverse Agonist for GPR12. Biochem. Biophys. Res. Commun..

[130] Kaplan J.S., Stella N., Catterall W.A., Westenbroek R.E. (2017). Cannabidiol Attenuates Seizures and Social Deficits in a Mouse Model of Dravet Syndrome. Proc. Natl. Acad. Sci. USA.

[131] Whyte L.S., Ryberg E., Sims N.A., Ridge S.A., Mackie K., Greasley P.J., Ross R.A., Rogers M.J. (2009). The Putative Cannabinoid Receptor GPR55 Affects Osteoclast Function in Vitro and Bone Mass In Vivo. Proc. Natl. Acad. Sci. USA.

[132] Ford L.A., Roelofs A.J., Anavi-Goffer S., Mowat L., Simpson D.G., Irving A.J., Rogers M.J., Rajnicek A.M., Ross R.A. (2010). A Role for L-α-Lysophosphatidylinositol and GPR55 in the Modulation of Migration, Orientation and Polarization of Human Breast Cancer Cells. Br. J. Pharmacol..

[133] Patricio F., Morales Dávila E., Patricio-Martínez A., Arana Del Carmen N., Martínez I., Aguilera J., Perez-Aguilar J.M., Limón I.D. (2022). Intrapallidal Injection of Cannabidiol or a Selective GPR55 Antagonist Decreases Motor Asymmetry and Improves Fine Motor Skills in Hemiparkinsonian Rats. Front. Pharmacol..

[134] Devinsky O., Nabbout R., Miller I., Laux L., Zolnowska M., Wright S., Roberts C. (2019). Long-term Cannabidiol Treatment in Patients with Dravet Syndrome: An Open-label Extension Trial. Epilepsia.

[135] Bisogno T., Hanuš L., De Petrocellis L., Tchilibon S., Ponde D.E., Brandi I., Moriello A.S., Davis J.B., Mechoulam R., Di Marzo V. (2001). Molecular Targets for Cannabidiol and Its Synthetic Analogues: Effect on Vanilloid VR1 Receptors and on the Cellular Uptake and Enzymatic Hydrolysis of Anandamide. Br. J. Pharmacol..

[136] Murillo-Rodriguez E., Blanco-Centurion C., Sanchez C., Daniele P., Shiromani P.J. (2003). Anandamide Enhances Extracellular Levels of Adenosine and Induces Sleep: An In Vivo Microdialysis Study. Sleep.

[137] Mayo L.M., Asratian A., Lindé J., Morena M., Haataja R., Hammar V., Augier G., Hill M.N., Heilig M. (2020). Elevated Anandamide, Enhanced Recall of Fear Extinction, and Attenuated Stress Responses Following Inhibition of Fatty Acid Amide Hydrolase: A Randomized, Controlled Experimental Medicine Trial. Biol. Psychiatry.

[138] Wang Y., Zhang X. (2017). FAAH Inhibition Produces Antidepressant-like Efforts of Mice to Acute Stress via Synaptic Long-Term Depression. Behav. Brain Res..

[139] Russo E.B., Burnett A., Hall B., Parker K.K. (2005). Agonistic Properties of Cannabidiol at 5-HT1a Receptors. Neurochem. Res..

[140] Rock E., Bolognini D., Limebeer C., Cascio M., Anavi-Goffer S., Fletcher P., Mechoulam R., Pertwee R., Parker L. (2012). Cannabidiol, a Non-Psychotropic Component of Cannabis, Attenuates Vomiting and Nausea-like Behaviour via Indirect Agonism of 5-HT1A Somatodendritic Autoreceptors in the Dorsal Raphe Nucleus. Br. J. Pharmacol..

[141] Jesus C.H.A., Redivo D.D.B., Gasparin A.T., Sotomaior B.B., de Carvalho M.C., Genaro K., Zuardi A.W., Hallak J.E.C., Crippa J.A., Zanoveli J.M. (2019). Cannabidiol Attenuates Mechanical Allodynia in Streptozotocin-Induced Diabetic Rats via Serotonergic System Activation through 5-HT1A Receptors. Brain Res..

[142] Martínez-Aguirre C., Carmona-Cruz F., Velasco A.L., Velasco F., Aguado-Carrillo G., Cuéllar-Herrera M., Rocha L. (2020). Cannabidiol Acts at 5-HT1A Receptors in the Human Brain: Relevance for Treating Temporal Lobe Epilepsy. Front. Behav. Neurosci..

[143] Fogaça M.V., Reis F.M.C.V., Campos A.C., Guimarães F.S. (2014). Effects of Intra-Prelimbic Prefrontal Cortex Injection of Cannabidiol on Anxiety-like Behavior: Involvement of 5HT1A Receptors and Previous Stressful Experience. Eur. Neuropsychopharmacol..

[144] Gomes F.V., Resstel L.B.M., Guimarães F.S. (2011). The Anxiolytic-like Effects of Cannabidiol Injected into the Bed Nucleus of the Stria Terminalis Are Mediated by 5-HT1A Receptors. Psychopharmacology.

[145] Linge R., Jiménez-Sánchez L., Campa L., Pilar-Cuéllar F., Vidal R., Pazos A., Adell A., Díaz Á. (2016). Cannabidiol Induces Rapid-Acting Antidepressant-like Effects and Enhances Cortical 5-HT/Glutamate Neurotransmission: Role of 5-HT1A Receptors. Neuropharmacology.

[146] Zanelati T., Biojone C., Moreira F., Guimarães F., Joca S. (2010). Antidepressant-like Effects of Cannabidiol in Mice: Possible Involvement of 5-HT1A Receptors. Br. J. Pharmacol..

[147] Ward S.J., McAllister S.D., Kawamura R., Murase R., Neelakantan H., Walker E.A. (2014). Cannabidiol Inhibits Paclitaxel-Induced Neuropathic Pain through 5-HT 1A Receptors without Diminishing Nervous System Function or Chemotherapy Efficacy. Br. J. Pharmacol..

[148] Rodrigues da Silva N., Gomes F.V., Sonego A.B., Silva N.R.d., Guimarães F.S. (2020). Cannabidiol Attenuates Behavioral Changes in a Rodent Model of Schizophrenia through 5-HT1A, but Not CB1 and CB2 Receptors. Pharmacol. Res..

[149] Resstel L.B.M., Tavares R.F., Lisboa S.F.S., Joca S.R.L., Corrêa F.M.A., Guimarães F.S. (2009). 5-HT1A Receptors Are Involved in the Cannabidiol-Induced Attenuation of Behavioural and Cardiovascular Responses to Acute Restraint Stress in Rats. Br. J. Pharmacol..

[150] Rock E.M., Goodwin J.M., Limebeer C.L., Breuer A., Pertwee R.G., Mechoulam R., Parker L.A. (2011). Interaction between Non-Psychotropic Cannabinoids in Marihuana: Effect of Cannabigerol (CBG) on the Anti-Nausea or Anti-Emetic Effects of Cannabidiol (CBD) in Rats and Shrews. Psychopharmacology.

[151] Yang K.-H., Galadari S., Isaev D., Petroianu G., Shippenberg T.S., Oz M. (2010). The Nonpsychoactive Cannabinoid Cannabidiol Inhibits 5-Hydroxytryptamine3AReceptor-Mediated Currents in Xenopus Laevis Oocytes. J. Pharmacol. Exp. Ther..

[152] Kossakowski R., Schlicker E., Toczek M., Weresa J., Malinowska B. (2019). Cannabidiol Affects the Bezold-Jarisch Reflex via TRPV1 and 5-HT3 Receptors and Has Peripheral Sympathomimetic Effects in Spontaneously Hypertensive and Normotensive Rats. Front. Pharmacol..

[153] Gonca E., Darıcı F. (2015). The Effect of Cannabidiol on Ischemia/Reperfusion-Induced Ventricular Arrhythmias. J. Cardiovasc. Pharmacol. Ther..

[154] Maione S., Piscitelli F., Gatta L., Vita D., De Petrocellis L., Palazzo E., De Novellis V., Di Marzo V. (2011). Non-Psychoactive Cannabinoids Modulate the Descending Pathway of Antinociception in Anaesthetized Rats through Several Mechanisms of Action. Br. J. Pharmacol..

[155] Carrier E.J., Auchampach J.A., Hillard C.J. (2006). Inhibition of an Equilibrative Nucleoside Transporter by Cannabidiol: A Mechanism of Cannabinoid Immunosuppression. Proc. Natl. Acad. Sci. USA.

[156] Magen I., Avraham Y., Ackerman Z., Vorobiev L., Mechoulam R., Berry E.M. (2009). Cannabidiol Ameliorates Cognitive and Motor Impairments in Mice with Bile Duct Ligation. J. Hepatol..

[157] Mecha M., Feliú A., Iñigo P.M., Mestre L., Carrillo-Salinas F.J., Guaza C. (2013). Cannabidiol Provides Long-Lasting Protection against the Deleterious Effects of Inflammation in a Viral Model of Multiple Sclerosis: A Role for A2A Receptors. Neurobiol. Dis..

[158] Esposito G., Scuderi C., Valenza M., Togna G.I., Latina V., De Filippis D., Cipriano M., Carratù M.R., Iuvone T., Steardo L. (2011). Cannabidiol Reduces Aβ-Induced Neuroinflammation and Promotes Hippocampal Neurogenesis through PPARγ Involvement. PLoS ONE.

[159] Nichols J.M., Kaplan B.L.F. (2020). Immune Responses Regulated by Cannabidiol. Cannabis Cannabinoid Res..

[160] Malfait A.M., Gallily R., Sumariwalla P.F., Malik A.S., Andreakos E., Mechoulam R., Feldmann M. (2000). The Nonpsychoactive Cannabis Constituent Cannabidiol Is an Oral Anti-Arthritic Therapeutic in Murine Collagen-Induced Arthritis. Proc. Natl. Acad. Sci. USA.

[161] Kozela E., Lev N., Kaushansky N., Eilam R., Rimmerman N., Levy R., Ben-Nun A., Juknat A., Vogel Z. (2011). Cannabidiol Inhibits Pathogenic T Cells, Decreases Spinal Microglial Activation and Ameliorates Multiple Sclerosis-like Disease in C57BL/6 Mice. Br. J. Pharmacol..

[162] Ahrens J., Demir R., Leuwer M., de la Roche J., Krampfl K., Foadi N., Karst M., Haeseler G. (2009). The Nonpsychotropic Cannabinoid Cannabidiol Modulates and Directly Activates Alpha-1 and Alpha-1-Beta Glycine Receptor Function. Pharmacology.

[163] Xiong W., Cui T., Cheng K., Yang F., Chen S.-R., Willenbring D., Guan Y., Pan H.-L., Ren K., Xu Y. (2012). Cannabinoids Suppress Inflammatory and Neuropathic Pain by Targeting A3 Glycine Receptors. J. Exp. Med..

[164] Bakas T., van Nieuwenhuijzen P.S., Devenish S.O., McGregor I.S., Arnold J.C., Chebib M. (2017). The Direct Actions of Cannabidiol and 2-Arachidonoyl Glycerol at GABA A Receptors. Pharmacol. Res..

[165] Iannotti F.A., Hill C.L., Leo A., Alhusaini A., Soubrane C., Mazzarella E., Russo E., Whalley B.J., Di Marzo V., Stephens G.J. (2014). Nonpsychotropic Plant Cannabinoids, Cannabidivarin (CBDV) and Cannabidiol (CBD), Activate and Desensitize Transient Receptor Potential Vanilloid 1 (TRPV1) Channels in Vitro: Potential for the Treatment of Neuronal Hyperexcitability. ACS Chem. Neurosci..

[166] Campos A.C., Guimarães F.S. (2009). Evidence for a Potential Role for TRPV1 Receptors in the Dorsolateral Periaqueductal Gray in the Attenuation of the Anxiolytic Effects of Cannabinoids. Prog. Neuropsychopharmacol. Biol. Psychiatry.

[167] Fonseca B.M., Correia-da-Silva G., Teixeira N.A. (2018). Cannabinoid-Induced Cell Death in Endometrial Cancer Cells: Involvement of TRPV1 Receptors in Apoptosis. J. Physiol. Biochem..

[168] Hassan S., Eldeeb K., Millns P.J., Bennett A.J., Alexander S.P.H., Kendall D.A. (2014). Cannabidiol Enhances Microglial Phagocytosis via Transient Receptor Potential (TRP) Channel Activation. Br. J. Pharmacol..

[169] Seeman P. (2016). Cannabidiol Is a Partial Agonist at Dopamine D2High Receptors, Predicting Its Antipsychotic Clinical Dose. Transl. Psychiatry.

[170] Navarro G., Varani K., Reyes-Resina I., de Medina V.S., Rivas-Santisteban R., Callado C.S.C., Vincenzi F., Casano S., Ferreiro-Vera C., Canela E.I. (2018). Cannabigerol Action at Cannabinoid CB1 and CB2 Receptors and at CB_1_–CB_2_ Heteroreceptor Complexes. Front. Pharmacol..

[171] Borrelli F., Fasolino I., Romano B., Capasso R., Maiello F., Coppola D., Orlando P., Battista G., Pagano E., Di Marzo V. (2013). Beneficial Effect of the Non-Psychotropic Plant Cannabinoid Cannabigerol on Experimental Inflammatory Bowel Disease. Biochem. Pharmacol..

[172] Cascio M., Gauson L., Stevenson L., Ross R., Pertwee R. (2010). Evidence That the Plant Cannabinoid Cannabigerol Is a Highly Potent A2-Adrenoceptor Agonist and Moderately Potent 5HT1A Receptor Antagonist. Br. J. Pharmacol..

[173] Borrelli F., Pagano E., Romano B., Panzera S., Maiello F., Coppola D., De Petrocellis L., Buono L., Orlando P., Izzo A.A. (2014). Colon Carcinogenesis Is Inhibited by the TRPM8 Antagonist Cannabigerol, a Cannabis-Derived Non-Psychotropic Cannabinoid. Carcinogenesis.

[174] Udoh M., Santiago M., Devenish S., McGregor I.S., Connor M. (2019). Cannabichromene Is a Cannabinoid CB2 Receptor Agonist. Br. J. Pharmacol..

[175] Romano B., Borrelli F., Fasolino I., Capasso R., Piscitelli F., Cascio M.G., Pertwee R.G., Coppola D., Vassallo L., Orlando P. (2013). The Cannabinoid TRPA1 Agonist Cannabichromene Inhibits Nitric Oxide Production in Macrophages and Ameliorates Murine Colitis. Br. J. Pharmacol..

[176] Rhee M.-H., Vogel Z., Barg J., Bayewitch M., Levy R., Hanuš L., Breuer A., Mechoulam R. (1997). Cannabinol Derivatives: Binding to Cannabinoid Receptors and Inhibition of Adenylylcyclase. J. Med. Chem..

[177] Farrimond J.A., Whalley B.J., Williams C.M. (2012). Cannabinol and Cannabidiol Exert Opposing Effects on Rat Feeding Patterns. Psychopharmacology.

[178] MacLennan S.J., Reynen P.H., Kwan J., Bonhaus D.W. (1998). Evidence for Inverse Agonism of SR141716A at Human Recombinant Cannabinoid CB1 and CB2 Receptors. Br. J. Pharmacol..

[179] Thomas A., Stevenson L.A., Wease K.N., Price M.R., Baillie G., Ross R.A., Pertwee R.G. (2005). Evidence That the Plant Cannabinoid Δ^9^-Tetrahydrocannabivarin Is a Cannabinoid CB1 and CB2 Receptor Antagonist. Br. J. Pharmacol..

[180] Pertwee R.G., Thomas A., Stevenson L.A., Ross R.A., Varvel S.A., Lichtman A.H., Martin B.R., Razdan R.K. (2007). The Psychoactive Plant Cannabinoid, Δ^9^-Tetrahydrocannabinol, Is Antagonized by Δ8- and Δ9-Tetrahydrocannabivarin in Mice In Vivo. Br. J. Pharmacol..

[181] Bolognini D., Costa B., Maione S., Comelli F., Marini P., Di Marzo V., Parolaro D., Ross R.A., Gauson L.A., Cascio M.G. (2010). The Plant Cannabinoid Δ^9^-Tetrahydrocannabivarin Can Decrease Signs of Inflammation and Inflammatory Pain in Mice. Br. J. Pharmacol..

[182] Hill A.J., Weston S.E., Jones N.A., Smith I., Bevan S.A., Williamson E.M., Stephens G.J., Williams C.M., Whalley B.J. (2010). Δ^9^-Tetrahydrocannabivarin Suppresses in Vitro Epileptiform and In Vivo Seizure Activity in Adult Rats. Epilepsia.

[183] Riedel G., Fadda P., McKillop-Smith S., Pertwee R.G., Platt B., Robinson L. (2009). Synthetic and Plant-Derived Cannabinoid Receptor Antagonists Show Hypophagic Properties in Fasted and Non-Fasted Mice. Br. J. Pharmacol..

[184] Wargent E.T., Zaibi M.S., Silvestri C., Hislop D.C., Stocker C.J., Stott C.G., Guy G.W., Duncan M., Di Marzo V., Cawthorne M.A. (2013). The Cannabinoid Δ^9^-Tetrahydrocannabivarin (THCV) Ameliorates Insulin Sen-sitivity in Two Mouse Models of Obesity. Nutr. Diabetes.

[185] Jadoon K.A., Ratcliffe S.H., Barrett D.A., Thomas E.L., Stott C., Bell J.D., O’Sullivan S.E., Tan G.D. (2016). Effica-cy and Safety of Cannabidiol and Tetrahydrocannabivarin on Glycemic and Lipid Parameters in Patients with Type 2 Diabetes: A Randomized, Double-Blind, Placebo-Controlled, Parallel Group Pilot Study. Diabetes Care.

[186] Cascio M.G., Zamberletti E., Marini P., Parolaro D., Pertwee R.G. (2015). The Phytocannabinoid, Δ^9^-Tetrahydrocannabivarin, Can Act through 5-HT1A Receptors to Produce Antipsychotic Effects. Br. J. Pharmacol..

[187] Morano A., Cifelli P., Nencini P., Antonilli L., Fattouch J., Ruffolo G., Roseti C., Aronica E., Limatola C., Di Bonaven-tura C. (2016). Cannabis in Epilepsy: From Clinical Practice to Basic Research Focusing on the Possible Role of Cannabidivarin. Epilepsia Open.

[188] Huizenga M.N., Sepulveda-Rodriguez A., Forcelli P.A. (2019). Preclinical Safety and Efficacy of Cannabidivarin for Early Life Seizures. Neuropharmacology.

[189] Palomares B., Ruiz-Pino F., Garrido-Rodriguez M., Eugenia Prados M., Sánchez-Garrido M.A., Velasco I., Vazquez M.J., Nadal X., Ferreiro-Vera C., Morrugares R. (2020). Tetrahydrocannabinolic Acid A (THCA-A) Reduces Adiposity and Prevents Metabolic Disease Caused by Diet-Induced Obesity. Biochem. Pharmacol..

[190] Nadal X., del Río C., Casano S., Palomares B., Ferreiro-Vera C., Navarrete C., Sánchez-Carnerero C., Can-tarero I., Bellido M.L., Meyer S. (2017). Tetrahydrocannabinolic Acid Is a Potent PPARγ Agonist with Neu-roprotective Activity. Br. J. Pharmacol..

[191] Bolognini D., Rock E., Cluny N., Cascio M., Limebeer C., Duncan M., Stott C., Javid F., Parker L., Pertwee R. (2013). Cannabidiolic Acid Prevents Vomiting in Suncus Murinus and Nausea-Induced Behaviour in Rats by Enhancing 5-HT1A Receptor Activation. Br. J. Pharmacol..

[192] Anderson L.L., Low I.K., Banister S.D., McGregor I.S., Arnold J.C. (2019). Pharmacokinetics of Phytocannabinoid Acids and Anticonvulsant Effect of Cannabidiolic Acid in a Mouse Model of Dravet Syndrome. J. Nat. Prod..

[193] Pertwee R.G., Rock E.M., Guenther K., Limebeer C.L., Stevenson L.A., Haj C., Smoum R., Parker L.A., Mechoulam R. (2018). Cannabidiolic Acid Methyl Ester, a Stable Synthetic Analogue of Cannabidiolic Acid, Can Produce 5-HT1A Receptor-Mediated Suppression of Nausea and Anxiety in Rats. Br. J. Pharmacol..

[194] Huffman J.W., Liddle J., Yu S., Aung M.M., Abood M.E., Wiley J.L., Martin B.R. (1999). 3-(1′,1′-Dimethylbutyl)-1-Deoxy-Δ^8^-THC and Related Compounds: Synthesis of Selective Ligands for the CB2 Receptor. Bioorg. Med. Chem..

[195] Avraham Y., Ben-Shushan D., Breuer A., Zolotarev O., Okon A., Fink N., Katz V., Berry E.M. (2004). Very Low Doses of Δ^8^-THC Increase Food Consumption and Alter Neurotransmitter Levels Following Weight Loss. Pharmacol. Biochem. Behav..

[196] Stasiulewicz A., Znajdek K., Grudzień M., Pawiński T., Sulkowska J.I. (2020). A Guide to Targeting the Endocannabinoid System in Drug Design. Int. J. Mol. Sci..

[197] de Almeida D.L., Devi L.A. (2020). Diversity of Molecular Targets and Signaling Pathways for CBD. Pharmacol. Res. Perspect..

[198] Oultram J.M.J., Pegler J.L., Bowser T.A., Ney L.J., Eamens A.L., Grof C.P.L. (2021). *Cannabis Sativa*: Interdisciplinary Strategies and Avenues for Medical and Commercial Progression Outside of CBD and THC. Biomedicines.

[199] Peng J., Fan M., An C., Ni F., Huang W., Luo J. (2022). A Narrative Review of Molecular Mechanism and Therapeutic Effect of Cannabidiol (CBD). Basic Clin. Pharmacol. Toxicol..

[200] Matheson J., Bourgault Z., Le Foll B. (2022). Sex Differences in the Neuropsychiatric Effects and Pharmacokinetics of Cannabidiol: A Scoping Review. Biomolecules.

[201] Gertsch J., Leonti M., Raduner S., Racz I., Chen J.-Z., Xie X.-Q., Altmann K.-H., Karsak M., Zimmer A. (2008). Beta-Caryophyllene Is a Dietary Cannabinoid. Proc. Natl. Acad. Sci. USA.

[202] Gertsch J. (2008). Antiinflammatory Cannabinoids in Diet–towards a Better Understanding of CB2 Receptor Action?. Commun. Integr. Biol..

[203] Aly E., Khajah M.A., Masocha W. (2020). β-Caryophyllene, a CB2-Receptor-Selective Phytocannabinoid, Suppresses Mechanical Allodynia in a Mouse Model of Antiretroviral-Induced Neuropathic Pain. Molecules.

[204] Varga Z.V., Matyas C., Erdelyi K., Cinar R., Nieri D., Chicca A., Nemeth B.T., Paloczi J., Lajtos T., Corey L. (2018). β-Caryophyllene Protects against Alcoholic Steatohepatitis by Attenuating Inflammation and Metabolic Dysregulation in Mice. Br. J. Pharmacol..

[205] Katsuyama S., Mizoguchi H., Kuwahata H., Komatsu T., Nagaoka K., Nakamura H., Bagetta G., Sakurada T., Sakurada S. (2013). Involvement of Peripheral Cannabinoid and Opioid Receptors in β-Caryophyllene-Induced Antinociception. Eur. J. Pain.

[206] Segat G.C., Manjavachi M.N., Matias D.O., Passos G.F., Freitas C.S., Costa R., Calixto J.B. (2017). Antiallodynic Effect of β-Caryophyllene on Paclitaxel-Induced Peripheral Neuropathy in Mice. Neuropharmacology.

[207] Scandiffio R., Bonzano S., Cottone E., Shrestha S., Bossi S., De Marchis S., Maffei M.E., Bovolin P. (2023). Beta-Caryophyllene Modifies Intracellular Lipid Composition in a Cell Model of Hepatic Steatosis by Acting through CB2 and PPAR Receptors. Int. J. Mol. Sci..

[208] Fidyt K., Fiedorowicz A., Strządała L., Szumny A. (2016). β-Caryophyllene and β-Caryophyllene Oxide-Natural Compounds of Anticancer and Analgesic Properties. Cancer Med..

[209] Tian X., Liu H., Xiang F., Xu L., Dong Z. (2019). β-Caryophyllene Protects against Ischemic Stroke by Promoting Polarization of Microglia toward M2 Phenotype via the TLR4 Pathway. Life Sci..

[210] Yang M., Lv Y., Tian X., Lou J., An R., Zhang Q., Li M., Xu L., Dong Z. (2017). Neuroprotective Effect of β-Caryophyllene on Cerebral Ischemia-Reperfusion Injury via Regulation of Necroptotic Neuronal Death and Inflammation: In Vivo and in Vitro. Front. Neurosci..

[211] Komiya M., Takeuchi T., Harada E. (2006). Lemon Oil Vapor Causes an Anti-Stress Effect via Modulating the 5-HT and DA Activities in Mice. Behav. Brain Res..

[212] Kaimoto T., Hatakeyama Y., Takahashi K., Imagawa T., Tominaga M., Ohta T. (2016). Involvement of Transient Receptor Potential A1 Channel in Algesic and Analgesic Actions of the Organic Compound Limonene. Eur. J. Pain.

[213] Piccinelli A.C., Morato P.N., dos Santos Barbosa M., Croda J., Sampson J., Kong X., Konkiewitz E.C., Ziff E.B., Amaya-Farfan J., Kassuya C.A.L. (2017). Limonene Reduces Hyperalgesia Induced by Gp120 and Cytokines by Modulation of IL-1 β and Protein Expression in Spinal Cord of Mice. Life Sci..

[214] d’Alessio P.A., Ostan R., Bisson J.-F., Schulzke J.D., Ursini M.V., Béné M.C. (2013). Oral Administration of D-Limonene Controls Inflammation in Rat Colitis and Displays Anti-Inflammatory Properties as Diet Supplementation in Humans. Life Sci..

[215] Park H.M., Lee J.H., Yaoyao J., Jun H.J., Lee S.J. (2011). Limonene, a Natural Cyclic Terpene, Is an Agonistic Ligand for Adenosine A2A Receptors. Biochem. Biophys. Res. Commun..

[216] Vigushin D.M., Poon G.K., Boddy A., English J., Halbert G.W., Pagonis C., Jarman M., Coombes R.C. (1998). Phase I and Pharmacokinetic Study of d -Limonene in Patients with Advanced Cancer. Cancer Chemother. Pharmacol..

[217] Kim D.-S., Lee H.-J., Jeon Y.-D., Han Y.-H., Kee J.-Y., Kim H.-J., Shin H.-J., Kang J., Lee B.S., Kim S.-H. (2015). Alpha-Pinene Exhibits Anti-Inflammatory Activity Through the Suppression of MAPKs and the NF-ΚB Pathway in Mouse Peritoneal Macrophages. Am. J. Chin. Med..

[218] Jo H., Cha B., Kim H., Brito S., Kwak B.M., Kim S.T., Bin B.-H., Lee M.-G. (2021). α-Pinene Enhances the Anti-cancer Activity of Natural Killer Cells via ERK/AKT Pathway. Int. J. Mol. Sci..

[219] Astani A., Reichling J., Schnitzler P. (2010). Comparative Study on the Antiviral Activity of Selected Monoterpenes Derived from Essential Oils. Phytother. Res..

[220] Jansen C., Shimoda L.M.N., Kawakami J.K., Ang L., Bacani A.J., Baker J.D., Badowski C., Speck M., Stokes A.J., Small-Howard A.L. (2019). Myrcene and Terpene Regulation of TRPV1. Channels.

[221] Rao V.S.N., Menezes A.M.S., Viana G.S.B. (2011). Effect of Myrcene on Nociception in Mice. J. Pharm. Pharmacol..

[222] Peana A.T., Rubattu P., Piga G.G., Fumagalli S., Boatto G., Pippia P., De Montis M.G. (2006). Involvement of Adenosine A1 and A2A Receptors in (−)-Linalool-Induced Antinociception. Life Sci..

[223] Harada H., Kashiwadani H., Kanmura Y., Kuwaki T. (2018). Linalool Odor-Induced Anxiolytic Effects in Mice. Front. Behav. Neurosci..

[224] Han H.D., Cho Y.-J., Cho S.K., Byeon Y., Jeon H.N., Kim H.-S., Kim B.-G., Bae D.-S., Lopez-Berestein G., Sood A.K. (2016). Linalool-Incorporated Nanoparticles as a Novel Anticancer Agent for Epithelial Ovarian Carcinoma. Mol. Cancer Ther..

[225] Gonçalves E.C.D., Baldasso G.M., Bicca M.A., Paes R.S., Capasso R., Dutra R.C. (2020). Terpenoids, Cannabimimetic Ligands, beyond the Cannabis Plant. Molecules.

[226] Liktor-Busa E., Keresztes A., Lavigne J., Streicher J.M., Largent-Milnes T.M. (2021). Analgesic Potential of Terpenes Derived from *Cannabis Sativa*. Pharmacol. Rev..

